# Synthesis, Structural Investigations, Molecular Docking, and Anticancer Activity of Some Novel Schiff Bases and Their Uranyl Complexes

**DOI:** 10.3390/biom11081138

**Published:** 2021-08-02

**Authors:** Hanan B. Howsaui, Amal S. Basaleh, Magda H. Abdellattif, Walid M. I. Hassan, Mostafa A. Hussien

**Affiliations:** 1 Department of Chemistry, Faculty of Science, King Abdulaziz University, P.O. Box 80203, Jeddah 21589, Saudi Arabia; hhowsaui0001@kau.edu.sa (H.B.H.); abasaleh@kau.edu.sa (A.S.B.); whassan@kau.edu.sa (W.M.I.H.); 2 Department of Chemistry, College of Sciences, Taif University, Al-Haweiah, P.O. Box 11099, Taif 21944, Saudi Arabia; 3 Department of Chemistry, Faculty of Science, Cairo University, Giza 12613, Egypt; 4 Department of Chemistry, Faculty of Science, Port Said University, Port Said 42521, Egypt

**Keywords:** uranyl complexes, Schiff base, DFT, molecular docking, DNA binding, anticancer

## Abstract

Three novel 2-aminopyrazine Schiff bases derived from salicylaldehyde derivatives and their uranyl complexes were synthesized and characterized by elemental analysis, UV-vis, FTIR, molar conductance, and thermal gravimetric analysis (TGA). The proposed structures were optimized using density functional theory (DFT/B3LYP) and 6–311G ∗(d,p) basis sets. All uranyl complexes are soluble in DMSO and have low molar conductance, which indicates that all the complexes are nonelectrolytes. The DNA binding of those Schiff bases and their uranyl complexes was studied using UV-vis spectroscopy, and screening of their ability to bind to calf thymus DNA (CT-DNA) showed that the complexes interact with CT-DNA through an intercalation mode, for which the K_b_ values ranged from 1 × 10^6^ to 3.33 × 10^5^ M^−1^. The anticancer activities of the Schiff base ligands and their uranyl complexes against two ovarian (Ovcar-3) and melanoma cell lines (M14) were investigated, and the results indicated that uranyl complexes exhibit better results than the Schiff base ligands. Molecular docking identified the distance, energy account, type, and position of links contributing to the interactions between these complexes and two different cancer proteins (3W2S and 2OPZ).

## 1. Introduction

Cancer is a major general health problem worldwide because it consists of a group of more than 100 different diseases. Cancer can develop almost anywhere in the body without warning. There is still a lack of research on methods of treatment because the disease has spread rapidly with human development and changing lifestyles. It is expected that deaths associated with cancer will increase by 2030 [1]. In recent years, research has predominantly focused on finding and improving cancer treatments. Ovarian cancer is one of the most common types of cancer in women. Epithelial ovarian cancer, a type of ovarian cancer, ranks as the fifth most prevalent cause of death in the world. Melanoma cancer, a type of skin cancer, is another of the most dangerous and common types. Infection rates are increasing globally because melanoma is linked to exposure to ultraviolet rays (UV) from sunlight or lamps and body tanning [2,3,4]. Schiff bases are organic compounds that have multiple biological applications and are easy to prepare by reactions of amines with carbonyl compounds. Schiff bases have been instrumental in medicine for a long time and are involved in the manufacture of pharmaceuticals. In many studies, Schiff bases and their metal complexes proved effective as antimicrobial, antifungal, antiviral, antioxidant, and anticancer agents [5,6,7] and for other applications involving biological activities. Uranyl Schiff base complexes, in particular, are of great value for different types of anticancer treatments and are reported as anticancer agents against Jurkat cell lines [8], HEPG-2 carcinoma cell lines [9], and Haman colon adenocarcinoma [10]. Most of the reported data about Uranyl complexes are about hepatic, breast, and colon carcinoma. In this study, the action and uses of uranyl complexes against M-14 melanoma and Ovcar-3 ovarian cell lines were illustrated. Uranyl ions have several different oxidation states, which explains the fact that they have attracted the attention of researchers during recent decades; there are many interesting aspects of uranyl chemistry, including reactivity, coordination behavior, bonding interactions with ligands, and possible applications. Over the years, Schiff base uranyl complexes have shown biological activity, such as anticancer [11,12,13], antimicrobial [12], and antibacterial [13] activities. In this study, synthesis, structural analysis, characterization, DFT, and biological studies were performed with Schiff bases and their uranyl complexes. The interaction of the complexes with CT-DNA was probed by employing UV–vis spectrophotometry. Molecular docking of the complexes was simulated with two types of cancer proteins. Finally, the anticancer activities of these complexes were investigated using two cancer cell lines (Ovcar-3 and M-14).

## 2. Experimental

### 2.1. Materials and Methods

High purity 2-Aminopyrazine, salicylaldehyde, and derivatives were purchased from Alfa Aesar and ACROS. Calf thymus DNA (CT-DNA) and uranyl acetate were purchased from Sigma-Aldrich.

IR spectra were recorded with a Bruker Alpha spectrophotometer in the range 400–4000 cm^−1^. UV-Vis spectra were recorded in the 200–800 nm range using a MultiSpec-1501 instrument. These spectra were obtained at room temperature with DMSO solutions, using matched 1.0 cm quartz cells. ^1^H (600 MHz) NMR spectra in DMSO-d^6^ solution were recorded on a Bruker Avance 600 MHz spectrometer, while ^13^C (125 MHz) NMR spectra in CDCl_3_ chemical shift (d) values were stated in parts per million (ppm) using internal standard tetramethylsilane. The D_2_O exchange confirmed the exchangeable protons (OH and NH), presented as *m*/*z*. High-resolution electrospray ionization (ESI) mass spectra were recorded using an Agilent Q-TOF 6520 instrument; all mass spectrometry results are reported as *m*/*z* values. A PerkinElmer TGA system was used to perform thermal analyses at temperatures up to 1000 °C in the air with a heating rate of 10 °C/min. Conductometric measurements were carried out by Metrohm 712, a conductometer equipped with a Haake D1 circulator. In a typical experiment, 10.0 mL of metal ion solution (5.0 × 10^−5^–1.0 × 10^−4^ mol L^−1^ was placed in the two-wall conductometer glass cell and the conductance of the solution was measured. A pH-metric adjustment was carried out with systronic-µ pH meter 361 having combined glass electrode and temperature probe maintained with readability ±0.1 °C. Theoretical DFT calculation in this work was performed at King Abdulaziz University’s High-Performance Computing Center (Aziz Super-computer) (http://hpc.kau.edu.sa, accessed on 29 May 2020).

### 2.2. Synthesis of Schiff Base Ligands

**Scheme 1:**
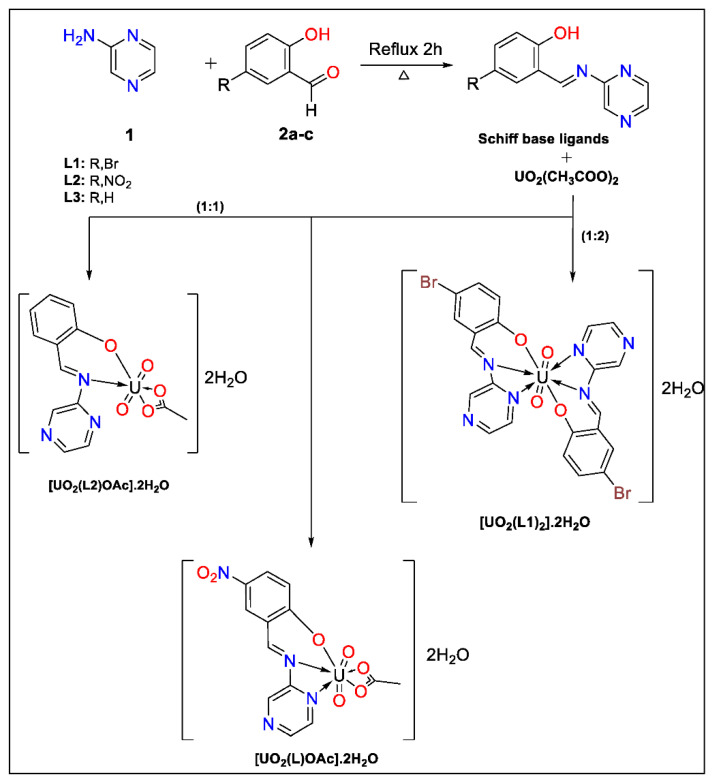
Synthesis of some novel Schiff bases and its uranyl complexes.

In a 100 mL round-bottom flask, a mixture of 1 mmol of 2-aminopyrazine and 1 mmol of substituted salicylaldehyde **2a–c** was dissolved in an ethanolic solution (99.9%) (50 mL). This reaction mixture was refluxed and heated to the boiling point for 2 h. Upon completion of the reaction, the product thus formed was filtered and washed with ethanol (Scheme 1).

i. (4-bromo-2-((pyrazin-2-ylimino)methyl)phenol) (L1)

Yield: 2.39 g, 82.91%, Color: Orange, Appearance: solid, M.p.: 149.6–151.4 °C. Anal. for C_11_H_8_BrN_3_O: C, 47.49; H, 2.85; N, 15.17; O, 5.70. Calc. C, 47.51; H, 2.90; N, 15.11; O, 5.75. FTIR (cm^−1^)(ν_CH=N_ azomethine)1602, (ν_C=N_ pyrazine ring) 1578.35, (ν_C-O_) 1252.98, (ν_C-Br_) 630.59. ^1^H NMR ((CD_3_)_2_SO) (δppm) 12.26 (bs.1H-OH). UV-Vis (DMSO) λ_max/_nm (cm^−^^1^) 266.16, 316.27 and 361.85 nm. MS *m/z* (%): 277.99 [M]^+^. Λ_M_ (10^−3^ M DMSO; ohm^−1^ cm^2^ mol^−1^); 14.

i. (4-nitro-2-((pyrazin-2-ylimino)methyl)phenol) (L2)

Yield: 2.4246 g, 93.12%, Color: Orange, Appearance: solid, M.p.: 210–212.7 °C. Anal. for C_11_H_8_N_4_O_3_ C, 53.89; H, 3.20; N, 22.90; O, 19.69. Calc. C, 54.10; H, 3.30; N, 22.94; O, 19.65. FTIR (cm^−1^) (ν_CH=N_ azomethine)1608.95, (ν_C=N_ pyrazine ring) 1578.35, (ν_C-O_) 1252.48, (ν_as C-NO__2_) 1559.70, (ν_s C-NO__2_) 1344.77. UV-vis (DMSO)λ_max/_nm (cm^−1^) 261.63 and 318.85 nm. MS *m/z* (%): 245.06 [M]^+^. Λ_M_(10^−3^ M DMSO; ohm^−1^ cm^2^ mol^−1^); 19.

(2-((pyraiz-2-ylimino)methyl)phenol) (L3)

Yield: 0.99 g, 47% Color: Orange, Appearance: solid, M.p.: 95–100.8 °C. Anal. for C_11_H_9_N_3_O C, 66.05; H,4.43; N, 21.14; O, 8.37. Calc. C, 66.32; H, 4.55; N, 21.09; O, 8.03. FTIR (cm^−1^) (ν_CH=N_ azomethine) 1602.98, (ν_C=N_ pyrazine ring) 1561.94, (ν_C-O_) 1252.98. ^1^H NMR ((CD_3_)_2_SO) (δppm) 12.38 (bs.1H-OH). UV-vis (DMSO)λ_max/_nm (cm^−1^) 254.28, 321.93 and 357.31 nm. MS *m/z* (%): 200.08 [M]^+^. Λ_M_(10^−3^ M DMSO; ohm^−1^ cm^2^ mol^−1^); 11.

### 2.3. Synthesis of Metal Complexes

#### 2.3.1. Metal Complexes of Schiff Base (1:2)

Schiff base ligand (L1) (2 mmol) was dissolved in 25 mL absolute ethanol. Drops of NaOH were added to a solution of 1 mmol of uranyl acetate in absolute ethanol until the pH reached 8. Then, the two solutions were combined and the heat was increased until the combined solution refluxed for 2 h. The complex was then filtered, washed with diethyl ether, and dried at room temperature.

i. Synthesis of [UO_2_(L1)_2_]·2H_2_O

Yield: 0.2468 g, 83.29%. Color: brown. Appearance: solid, M.p.: 122–320 °C. Anal. for C_22_H_14_Br_2_N_6_O_4_U C, 31.77; H, 1.69; N, 10.35; O, 7.80. Calc. C, 32.06; H, 1.71; Br, 19.39; N, 10.20; O, 7.76; U, 28.88. FTIR (cm^−1^) (ν_CH=N_ azomethine) 1639.91, (ν_C=N_ pyrazine ring) 1612.68, (ν_C-Br_) 624.62 (ν_U-N_) 468.34, (ν_U-O_) 548.50, (ν_H__2__O_) 3409.62. UV-vis (DMSO) λmax/nm (cm^−1^) 261.70, 304.25 and 402.69 nm. Λ_M_ (10^−3^ M DMSO; ohm^−1^ cm^2^ mol^−1^): 43.74. ^1^H NMR (600 MHz, DMSO-*d*_6_) δ 9.96 (s, 1H), 9.57 (s, 1H), 9.36 (dd, *J* = 4.7, 1.4 Hz, 1H), 9.16 (d, *J* = 4.7 Hz, 1H), 9.07–9.03 (m, 1H), 8.66–8.58 (m, 2H), 8.55–8.48 (m, 2H), 8.46 (dd, *J* = 7.9, 2.4 Hz, 1H), 7.89 (d, *J* = 7.7 Hz, 1H), 7.49 (d, *J* = 4.1 Hz, 1H), 7.28–7.21 (m, 2H), 5.22 (d, *J* = 4.6 Hz, 2H). ^13^C NMR (125 MHz, Chloroform-*d*) δ 166.48, 162.42, 155.25, 153.02, 152.12, 149.33, 148.58, 143.35, 138.12, 133.55, 133.39, 132.12, 129.97, 129.40, 127.47, 126.92, 124.12, 123.14 (d, *J* = 9.1 Hz), 115.42, 115.21.

#### 2.3.2. Metal Complexes of Schiff Bases (1:1)

Schiff base ligands (L2 and L3) (1 mmol) were dissolved in 25 mL absolute ethanol. Drops of NaOH were added to a solution of 1 mmol of uranyl acetate in absolute ethanol until the pH reached 8. Then, the two solutions were combined and the heat was increased until the combined solution refluxed for 2 h. The complex was then filtered, washed with diethyl ether, and dried at room temperature.

i. Synthesis of [UO_2_(L2)OAc]·2H_2_O

Yield: 0.3485 g, 88%. Color: yellow. Appearance: solid, M.p.: 291–321 °C. Anal. for C_13_H_14_N_4_O_9_U C, 25.59; H, 2.31; N, 9.14; O, 23.89. Calc. C, 25.67; H, 2.32; N, 9.21; O, 23.67; U, 39.13. FTIR (cm^−1^) (ν_CH=N_ azomethine) 1660.36, (ν_C=N_ pyrazine ring) 1601.49, (ν_U-N_) 465.76, (ν_U-O_) 512.12, (ν_H__2__O_) 3410.07. UV-vis (DMSO) λmax/nm (cm^−1^) 260, 373.09 and 432.14 nm. Λ_M_ (10^−3^ M DMSO; ohm^−1^ cm^2^ mol^−1^): 54.81. ^1^H NMR (500 MHz, DMSO-d_6_) δ 8.87 (d, J = 0.6 Hz, 1H), 8.68 (dd, J = 2.9, 1.4 Hz, 1H), 8.26 (dd, J = 7.5, 1.4 Hz, 1H), 7.71 (dd, J = 7.7, 1.1 Hz, 1H), 7.52 (td, J = 7.7, 1.4 Hz, 1H), 7.35 (td, J = 7.5, 1.1 Hz, 1H), 7.32–7.27 (m, 2H).

^13^C NMR (125 MHz, Chloroform-*d*) δ 156.40, 147.89 (d, *J* = 15.5 Hz), 144.95, 144.66, 143.91, 141.06, 129.44, 126.50, 125.21, 122.57 (d, *J* = 11.9 Hz), 13.40.

i. Synthesis of [UO_2_(L3)OAc]·2H_2_O

Yield:0.122 g, 69.6% Color: Light orange. M.p.: 204–390 °C. Anal. for C_13_H_16_N_3_O_5_U C, 27.65; H, 2.77; N, 7.69; O, 19.53. Calc. C, 27.67; H, 2.86; N, 7.45; O, 19.85; U, 42.18. FTIR (ν_CH=N_ azomethine) 1637.69, (ν_C=N_ pyrazine ring) 1558.95, (ν_U-N)_ 493.28, (ν_U-O_) 527.61, (ν_H__2__O_) 3414.45. UV-Vis (DMSO) λmax/nm (cm^−1^) 282.81, 355.68 and 402.93 nm. Λ_M_ (10^−3^ M DMSO; ohm^−1^ cm^2^ mol^−1^): 26.56. ^1^H NMR (500 MHz, DMSO-*d*_6_) δ 9.70–9.66 (m, 1H), 9.42 (dd, *J* = 4.9, 1.4 Hz, 1H), 9.28 (s, 1H), 9.15 (dd, *J* = 8.2, 2.1 Hz, 1H), 9.08 (d, *J* = 4.9 Hz, 1H), 8.26 (d, *J* = 8.3 Hz, 1H), 7.36 (d, *J* = 1.3 Hz, 1H).^13^C NMR (125 MHz, Chloroform-*d*) δ 161.68, 156.81, 153.32, 148.05, 145.01, 142.31, 142.13, 141.63, 130.00, 127.61, 125.61, 121.66, 13.86.

### 2.4. Mass Spectra

The ESI mass spectra of the Schiff base ligands recorded at room temperature were used to compare their stoichiometries and compositions. The fragmentation pattern of L1, given in Figures S1 and S2, presents the fragmentation pattern of L2, and Figure S3 illustrates the fragmentation pattern of L3.

### 2.5. Molar Ratio

The molar ratio of the metal ion was determined through Job’s method of equimolar solutions. This method depends on titration using a constant concentration of the metal ion. The concentration [M] = 0.72 ×10^−4^ M was used and this was pipetted into seven volumetric flasks (0, 1, 2, … 6 mL) and various ligand concentrations in the range [L] = 2.16 to 0.36 ×10^−4^ M, (6, 5, 4, … 0 mL) were added, as shown in Table S1. All measurements were made by a spectroscopic method in a 200–500 nm range [14,15,16].

### 2.6. Computational Details

All theoretical calculations were performed using the hybrid Becke 3 parameter functional B3LYP [17] implemented in the Gaussian 09 [18] software package. It is well known that the selection of the basis set affects the accuracy of the results. The uranyl acetate complex, which is very similar to the complexes under study, was used to validate the selection of the basis set. The validation of the basis set is summarized in Table 1. The % error was calculated against the reported experimental uranium oxygen bond length of 1.763 Å [19]. The data in Table 1 clearly show that the mixed basis set resulted in a large % error. Furthermore, the % error grew considerably as the size of the basis set increased. The results suggest the use of SDD [20] as a basis set for all atoms in this work. All the reported geometries were optimized using B3LYP/SDD in the ground state, and this was followed by frequency calculation and TDDFT to simulate the UV-Vis spectra and IR spectra of the studied complexes.

### 2.7. DNA Binding Studies

The DNA binding for ligands and their uranyl complexes was studied by the UV-Vis absorbance technique. The CT-DNA used was confirmed to be protein-free. All experiments were conducted at a pH value of 7.4 using Tris-HCl buffer. The Benesi–Hildebrand equation, Equation (1), and compensation with absorption values were used to calculate the binding constants (K_b_) of the ligands with CT-DNA. This equation is used for the study of the binding interactions of small molecules to macromolecules such as DNA.

(1) $$\frac{A^{0}}{\left(A-A^{0}\right)}=\frac{\varepsilon G}{\varepsilon \left(H-G\right)-\varepsilon G}+\frac{\varepsilon G}{(\varepsilon \left(H-G\right)-\varepsilon G}\frac{1}{K_{b}\left[\mathrm{DNA}\right]}$$

where [DNA] is the concentration of DNA and (A⁰) is the absorbance of a free compound. (A) is the absorbance of the compound in the presence of CT-DNA. (εG) and (εH-G) are molar absorptivities, and (K_b_) is the binding constant. ($\frac{A^{0}}{\left(A-A^{0}\right)}$) was plotted against$\;\left(\frac{1}{\left[\mathrm{DNA}\right]}\right)$ and (K_b_) was calculated from the ratio of intercept to the slope [21,22].

### 2.8. Molecular Docking

A program commonly used in molecular docking is the molecular operation environment (MOE). The crystal structures of target proteins for ovarian and melanoma cancer receptors were retrieved from the Protein Data Bank (http://www.rcsb.org/pdb/, accessed on 25 July 2020) (PDB codes: 3W2S and 2OPZ). These types were selected based on previous studies [23,24]. MOE was used to determine the modes of interaction between the protein receptor and complexes [25].

### 2.9. Anticancer and Toxicological Studies

Cell line screening was performed at the Faculty of Veterinary Science, Cairo University Central Laboratory. The following procedure was applied. The human tumor cell lines of the cancer screening panel were grown in RPMI 1640 medium containing 5% fetal bovine serum and 2 mM L-glutamine. For a typical screening experiment, 100 μL of cells were inoculated into 96-well microtiter plates at plating densities ranging from 5000 to 40,000 cells/well, depending on the doubling times of individual cell lines. After cell inoculation, the microtiter plates were incubated at 37 °C under 5% CO_2_, 95% air, and 100% relative humidity for 24 h before the addition of experimental drugs. After 24 h, two plates of each cell line were fixed in situ with TCA to represent a measurement of the cell population for each cell line at the time of drug addition (Tz). Experimental drugs are solubilized in dimethyl sulfoxide at a concentration 400-fold higher than the desired final maximum test concentration and stored frozen before use. At the time of drug addition, an aliquot of frozen concentrate was thawed and diluted to twice the desired final maximum test concentration with a complete medium containing 50 μg/mL gentamicin. An additional 4-fold, 10-fold, or ½log serial dilution was made to provide a total of five drug concentrations plus control. Aliquots of 100 μL of these different drug dilutions were added to the appropriate microtiter wells containing 100 μL of the medium, resulting in the required final drug concentrations. Following drug addition, the plates were incubated for an additional 48 h at 37 °C under 5% CO_2_, 95% air, and 100% relative humidity. For adherent cells, the assay was terminated by the addition of cold TCA. Cells were fixed in situ by the gentle addition of 50 μL of cold 50% (*w*/*v*) TCA (final concentration, 10% TCA) and incubated for 60 min at 4 °C. The supernatant was discarded, and the plates were washed five times with tap water and air-dried. Sulforhodamine B (SRB) solution (100 μL) at 0.4% (*w*/*v*) in 1% acetic acid was added to each well, and plates were incubated for 10 min at room temperature. After staining, the unbound dye was removed by washing five times with 1% acetic acid, and the plates were air-dried. The bound stain was subsequently solubilized with 10 mM Trizma base, and the absorbance was read on an automated plate reader at a wavelength of 515 nm. For suspended cells, the methodology was the same except that the assay was terminated by fixing settled cells at the bottom of the wells by gently adding 50 μL of 80% TCA (final concentration, 16% TCA). Using the seven absorbance measurements (time zero, (Tz), control growth, (C), and test growth in the presence of drug at the five concentration levels (Ti)), the percentage growth was calculated at each of the drug concentration levels. The percentage growth inhibition was calculated as $\left[\frac{\left(T_{i}-T_{Z}\right)}{\left(C-T_{Z}\right)}\right]\times 100$ for concentrations for which Ti ≥ Tz and $\left[\frac{\left(T_{i}-T_{Z}\right)}{\left(T_{Z}\right)}\right]\times 100$ for concentrations for which Ti < Tz. Three dose–response parameters were calculated for each experimental agent. Growth inhibition of 50% (IC50) was calculated from $\left[\frac{\left(T_{i}-T_{Z}\right)}{\left(C-T_{Z}\right)}\right]\times 100$ = 50, which is the drug concentration resulting in a 50% reduction in the net protein increase (as measured by SRB staining) in control cells during drug incubation. The drug concentration resulting in total growth inhibition (TGI) was calculated from Ti = Tz. The LC50 (concentration of drug resulting in a 50% reduction in the measured protein at the end of the drug treatment compared to that at the beginning), indicating a net loss of cells following treatment, was calculated from $\left[\frac{\left(T_{i}-T_{Z}\right)}{\left(T_{Z}\right)}\right]\times 100$ = −50. Values were calculated for each of these three parameters if the level of activity was reached; however, if the effect was not reached or was exceeded, the value for that parameter was expressed as greater or less than the maximum or minimum concentration tested.

## 3. Results and Discussion

### 3.1. IR Studies

To study the binding mode of Schiff base ligands to the central metal atom, the IR spectra of the free ligands were compared with the spectra of the complexes. The main IR bands and their assignments are listed in Table 2. The Schiff base ligand showed sharp (C=N) bands for the azomethine groups at 1602, 1608.95, and 1602.98 cm^−1^ for L1, L2, and L3, respectively [26]. After complexation, the vibration bands for the azomethine groups increased in intensity and were shifted towards lower energy by approximately 37–51 cm^−1^ in the uranyl complexes. This shift to a lower wavenumber or disappearance of peaks indicated complex formation. Another sharp (C=N) band for the pyrazine ring was observed. It appeared for the free ligand at 1578.35 cm^−1^, and this band shifted more than 5 cm^−1^ in the uranyl complexes, except for with [UO_2_(L3)OAc]·2H_2_O [27], which indicates that the pyrazine ring is involved in the coordination of metal ions in cases other than [UO_2_(L3)OAc]·2H_2_O. The ligands also displayed a band at 1252.98 cm^−1^, assigned to (C-O) stretching vibrations of phenolic-OH [28,29,30,31]. Finally, the (C-Br) band in L1 appeared at 630.59 cm^−1^ [32,33]. L2 exhibited two sharp peaks at 1559.70 (ν _asym_) and 1344.77 (ν _sym_) cm^−1^; these are due to the two (C-NO_2_) vibrations of the nitro groups [32], and they exhibited a slight displacement in the complexes.

Other new bands appeared in the ranges 900–840 cm^−1^ (ν_asym_) and 870–820 cm^−1^ (ν_sym_) for ν(O=U=O) of the uranyl complexes; new bands for ν(U-O) in the complexes appeared in the range 550–512 cm^−1^, the ν(U-N) band for the complexes appeared in the range 490–460 cm^−1^ [26], a new broad band at 3415–3409 cm^−1^, assigned to ν(H_2_O) Figure S4, appeared in the experimental IR spectra, and the calculated IR spectra are shown in Figure S5.

The uranyl ion radius was calculated by McGlynn’s equation (Equation (2)) and this was used to calculate the force constant (F_U-O_); the force constant is equal to (v_3_), the frequency for the asymmetric vibration of (O=U=O).

(2) $${\left(v_{3}\right)}^{2}=1307\times \frac{{\;F}_{U-O}}{14.103}$$

The calculated F_U-O_ values for the uranyl complexes were 6.653, 6.177, and 5.894 for [UO_2_(L1)_2_]·2H_2_O, [UO_2_(L2)OAc]·2H_2_O, and [UO_2_(L3)OAc]·2H_2_O, respectively. Additionally, Jones used Equation (3) to calculate the U-O bond length in Å, with $r_{U-O}$ as the uranyl radius.

(3) $$r_{U-O}=1.08\;{\left(F_{U-O}\right)}^{-\frac{1}{3}}+1.17$$

The results were 1.74, 1.75, and 1.76 Å cm^−1^ [26], and the data are given in Table 3.

Acetate vibrations for uranyl complexes [UO_2_(L2)_2_]·2H_2_O and [UO_2_(L3)OAc]·2H_2_O appeared for ν _asym_(COO^−^) at 1422.38 and 1410.83 cm^−1^ and for ν_sym_ (COO^−^) at 1367.16 cm^−1^ and 1338.50 cm^−1^, respectively. The nature of the acetate coordination to the metal may be determined by comparison with the value of ∆ free (for the free acetate ligand) with the value of ∆ complex for uranyl complexes, and ∆ free was calculated with Equation (4):

∆ free = (COO^−^)ν_asym_ − (COO^−^)ν_sym_ = 146 (4)

If ∆ free > ∆ complex, the coordination is bidentate and if ∆ complex > ∆ free, the coordination is monodentate [34,35,36].

The ∆ values for uranyl complexes were 55.22 and 72.33 for [UO_2_(L2)OAc]·2H_2_O and [UO_2_(L3)OAc]·2H_2_O, which indicate bidentate chelation of the acetate group.

### 3.2. ^1^H NMR Spectra for Ligands

**Figure 1:**
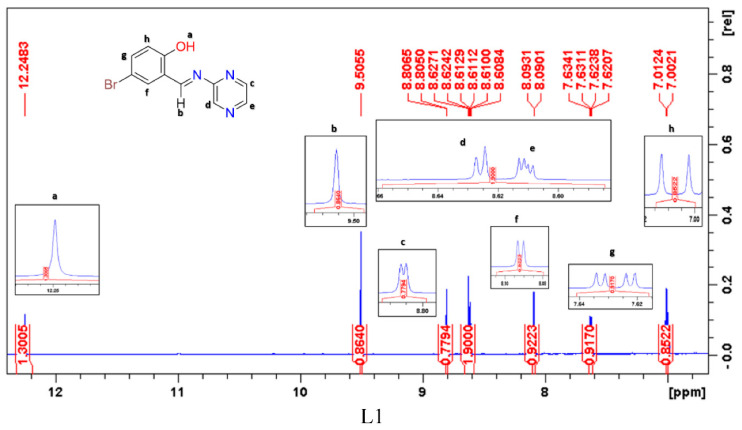
^1^H NMR spectra of L1.

The ^1^H NMR spectra of the Schiff bases showed singlets at 12.24, 10.30, and 12.38 δppm, indicating the presence of (phenolic-OH) protons [37,38], singlets at 9.53, 8.43, and 9.52 δppm, which can be ascribed to the azomethine proton (-CH=N) [39], singlets in the range 8.81–7.17 δppm, which indicate the presence of pyrazine protons, and singlets in the range of 7.65–6.81 δppm that can be ascribed to the aromatic benzene protons. The results for L1 are also depicted in Figure 1, and those for L2 and L3 are shown in Figure S6.

### 3.3. Conductance Measurements

The observed molar conductance values for ligands (Λ_M_ = 11–19) and uranyl complexes (Λ_M_ = 26.56- ohm^−1^ cm^2^ mol^−1^) in 10^−3^ M DMSO solutions are given in Table 4, and they suggest the nonelectrolyte nature of these compounds [40].

### 3.4. Molar Ratios

From the molar ratio studies, the molar ratio of the ligand to metal is 2:1 at inflection 0.33 for [UO_2_(L1)_2_]·2H_2_O and 1:1 for [UO_2_(L2)OAc]·2H_2_O and [UO_2_(L3)OAc]·2H_2_O complexes at inflection 0.5 at 25 °C, as shown in Figure S7.

### 3.5. The Electronic Spectra

Electronic spectra of all these complexes were determined in DMSO at 10^−3^ M. In the spectra of the Schiff base free ligands, maximal bands in the range 261 to 266 nm were due to π–π* transitions involving molecular orbitals located on phenolic chromophores, and bands between 316 and 360 nm were due to n–π* transitions of CH=N chromophores [41]; uranyl complexes showed absorption bands for π–π* and n–π* transitions, but they were shifted towards lower wavelengths (blueshifted), confirming the coordination of the ligands to metal ions [41]. Table S2 shows the experimental and calculated UV–vis spectra of the ligands and their uranyl complexes.

### 3.6. Thermal Analysis

**Figure 2:**
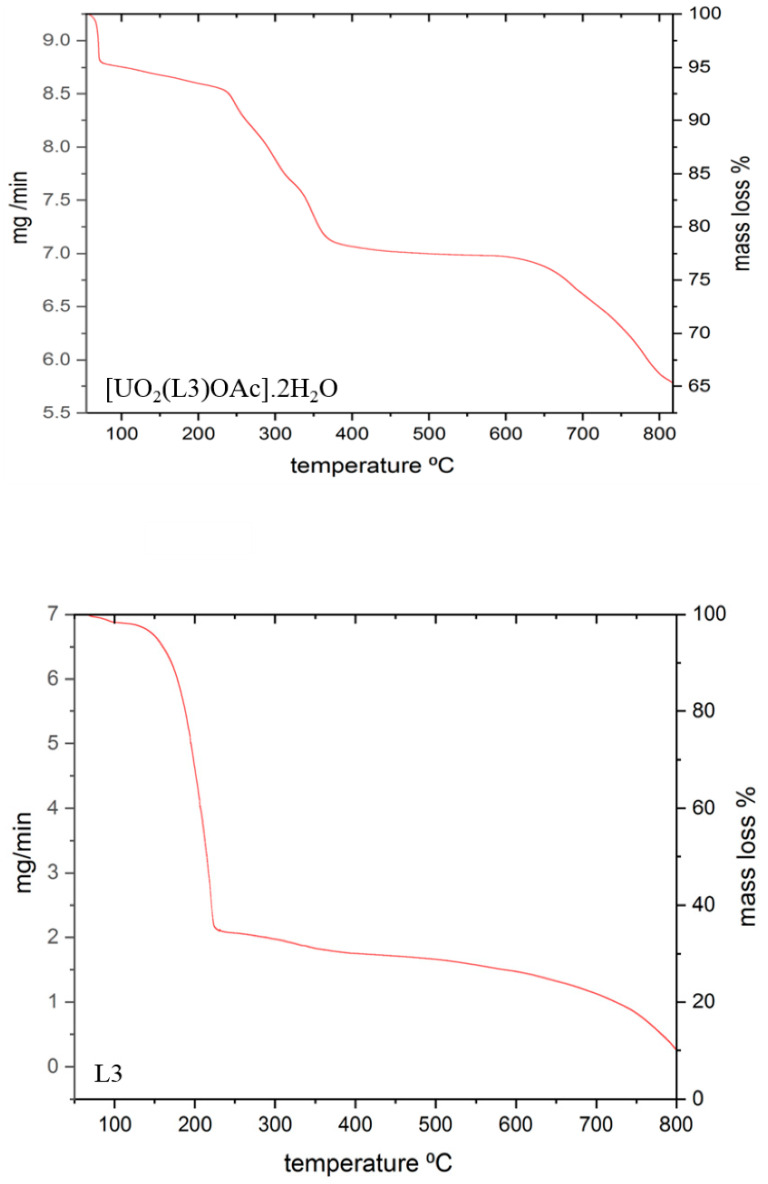
TG curves of L3 and [UO2(L3)OAc]·2H_2_O.

Thermal analyses of the ligands showed that they decompose in two steps in the temperature range 54–800 °C. These stages involved mass losses of 90.01% (90.31% calc.) for L1, 81.02% (80.96% calc.) for L2, and 90% (89.9% calc.) for L3. In the first step, these mass losses could be due to losses of the organic ligands as gases in the indicated temperature ranges; then, the next steps could correspond to the removal of the remaining organic part of the ligands [42]. Thermal analyses of the uranyl complexes indicated three decomposition steps that occurred in [UO_2_(L1)_2_]·2H_2_O and [UO_2_(L3)OAc]·2H_2_O and four decomposition steps for [UO_2_(L2)OAc]·2H_2_O [42]. The results of the decomposition steps for L1 are shown in Table 5. The results of the decomposition steps for the other compounds are shown in Table S4. TG curves for L3 and [UO_2_(L3)OAc]·2H_2_O are represented in Figure 2, and TG data for L1, L2, [UO_2_(L1)_2_]·2H_2_O, and [UO_2_(L2)OAc]·2H_2_O are shown in Figure S8.

The uranyl complex decomposition steps were as follows:

Removal of water molecules in the temperature range 54–128 °C, with mass losses of 22.7% (22.8% calc.) for [UO2(L1)2]·2H_2_O, 14.3% (14.2% calc.) for [UO2(L2)OAc]·2H_2_O and 5.5% (6.3% calc.) for [UO2(L3)OAc]·2H_2_O.

Decomposition of complexes due to the loss of organic moieties as gases in the temperature range 80–800 °C, with mass losses of 50.4% (50.8% calc.) for [UO2(L1)2]·2H_2_O, 50.5% (49.7% calc.) for [UO2(L2)OAc]·2H_2_O, and 29.5% (29.7% calc.) for [UO2(L3)OAc]·2H_2_O.

Loss of metallic residues of complexes at temperatures higher than 800 °C; (8C+O3U) for [UO2(L1)2]·2H_2_O, (UO3) for [UO2(L2)OAc]·2H_2_O, and (7C+O3U) for [UO2(L3)OAc]·2H_2_O.

### 3.7. Kinetic Studies

The thermodynamic activation parameters for the decomposition processes of the complexes, ΔH (enthalpy), ΔS (entropy), ΔG (Gibbs free energy change for the decomposition), and E (thermal activation energy), were determined by employing the Coast–Redfern (CR) and Horowitz–Metzger (HM) methods [43]. The results for L1 are shown in Table 6 and Table 7, and results for the remaining compounds are shown in Table S5. The results of Coast–Redfern and Horowitz–Metzger plots for the compounds are given in Table S6.

ΔH values > 0 indicate that the reaction is endothermic and endergonic because ΔG > 0; the positive values of ΔG denote that the reaction was nonspontaneous in the forward direction and spontaneous in the reverse direction [44].

### 3.8. Suggested Structures

**Figure 3:**
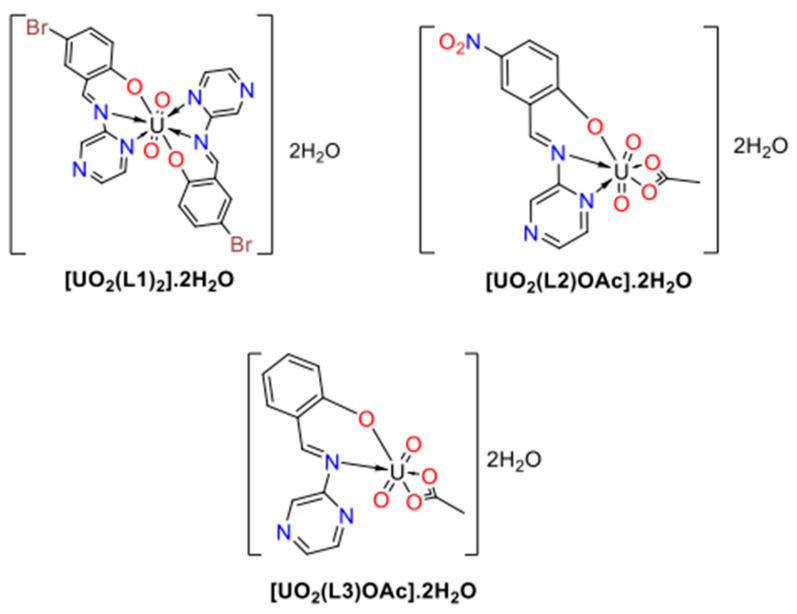
Suggested structure for the Uranyl complexes.

Based on the elemental analyses, IR and electronic spectra, molar ratio, molar conductance, and thermal analysis, the suggested structures for the three uranyl complexes are [UO_2_(L1)_2_]·2H_2_O [41], [UO_2_(L2)OAc]·2H_2_O (bipyramidal geometry) [45], and [UO_2_(L3)OAc]·2H_2_O [45]; these are shown in Figure 3 and have been tentatively proposed in the present study based on data from previous studies.

### 3.9. Computational Calculations

The geometries of the ligands and uranyl complexes were optimized completely. The relative stabilities of the compounds and their chemical reactivities were estimated by calculating quantum chemical parameters, and the results are shown in Table 8.

**Figure 4:**
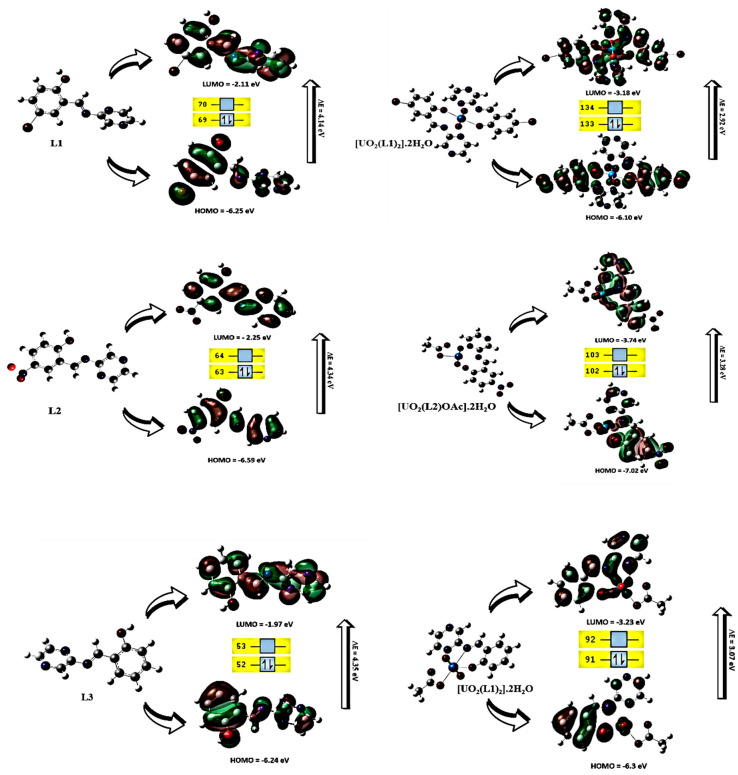
Homo and LUMO molecular orbitals.

The HOMO is the energy of the highest occupied molecular orbital and the LUMO is the energy of the lowest unoccupied molecular orbital, as shown in Figure 4. These values allowed calculation of quantum chemical parameters such as ΔE, the energy gap, by subtracting the HOMO energy from the LUMO energy; ΔE is an important indicator of the molecule’s stability. The greater the energy gap is, the more rigid, more stable, and less reactive the molecule. Absolute electronegativities (χ), absolute hardness (η), absolute softness (σ), chemical potentials (Pi), global softness (S), global electrophilicity (ω), and additional electronic charge (ΔN_max_) data are listed in Table 8. These results suggested that Schiff base ligands and their metal complexes are relatively stable. [46,47,48]

### 3.10. DNA-Binding Studies

**Figure 5:**
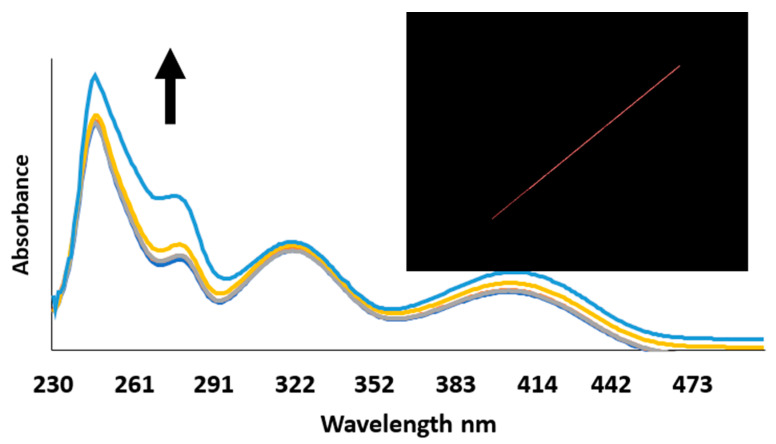
Absorption spectra of L1 in (Tris-HCl) buffer (pH = 7.4) at 25 °C with CT-DNA. The arrow indicates the increasing amount of DNA.

**Figure 6:**
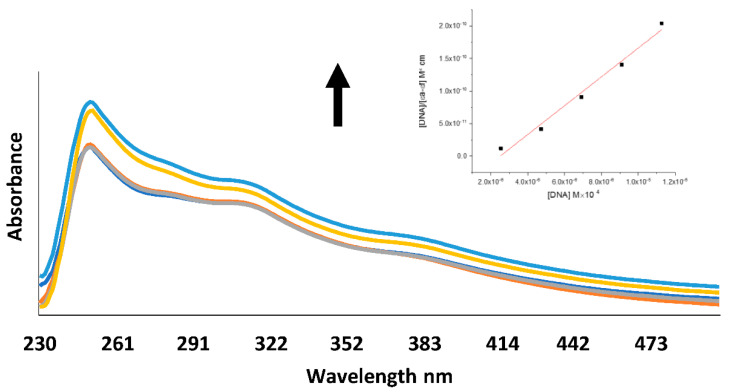
Absorption spectra of [UO_2_(L1)_2_]·2H_2_O in (Tris-HCl) buffer (pH = 7.4) at 25 °C with CT-DNA. The arrow indicates the increasing amount of DNA.

Electronic absorption titration is a common method used to predict the binding mode of compounds with CT-DNA [49]. In this study, the ligands and uranyl complexes exhibited one type of binding mode. They were identified by interpreting the spectra, and Figure 5 shows the absorption spectrum of L1. Figure 6 shows the absorption spectrum of [UO_2_(L1)_2_]·2H_2_O in Tris-HCl buffer (pH = 7.4) at 25 °C. Figures S9–S12 show the absorption spectra of L2, L3, [UO_2_(L2)OAc]·2H_2_O, and [UO_2_(L3)OAc]·2H_2_O, respectively.

Using data for the ligands and their uranyl complexes, it can be observed that with increasing DNA concentration, these compounds were hyperchromic. When comparing these complexes with the ligands, a blueshift in the charge transfer band occurred for these complexes. This indicated that the binding mode is intercalation, as reported in the literature [49]. The binding constants (K_b_) were calculated for each of the ligands and their complexes by preceding as in Equation (1) [21], and the results are summarized in Table 9.

All of the complexes gave (K_b_) values ranging from 1 × 10^−6^ to 3.33 × 10^−5^. By comparing these values with those for Ru complexes [50], the results suggest the binding mode is intercalation.

### 3.11. Molecular Docking

Molecular docking is a computational program routinely used for understanding drug-receptor interactions. We perform docking simulations on small molecule drug candidates, which we call ligands, and their target proteins inside the human body to predict how effective the ligand might be as a drug [16,51] After docking simulations, well-docked protein–ligand complexes are produced in experimental laboratories for testing. The docking process here was studied by simulating the coupling of the complexes with (M14) melanoma cancer cells (entry 2OPZ in the Protein Data Bank) and ovarian cancer cells (entry 3W2S in the Protein Data Bank), which were selected according to the literature and previous studies [52,53]. We aimed to determine the distance, energy account, type, and position of links contributing to the interaction between cancer proteins and ligands and their uranyl complexes. The docking study results are summarized in Table 10 and Table 11.

As the results show, uranyl complexes exhibited increases in their calculated energies as compared to those for the ligands. In addition, the highest binding energies for [UO_2_(L2)OAc]·2H_2_O were −6.73 Kcal mol^−1^ (with 3W2S protein) and −6.77 Kcal mol^−1^ (with 2OPZ protein), and these were due to H-donor, H-acceptor, and ionic interactions. Table 12 shows the best possible conformation inside the melanoma protein receptor (2OPZ) and ovarian protein receptor (3W2S) for the Schiff base ligands and uranyl complexes

### 3.12. Anticancer and Toxicological Studies

The ligands and their uranyl complexes were examined against two types of cancers. The results are shown in Table 13. The results clarified that uranyl complexes show better anticancer activities than ligands when both cells are examined [54,55,56,57,58,59].

By comparison with the literature results [8,9,10,11,12] of the activity of uranyl complexes as anticancer agents for many different cell lines, it was found that the Schiff’s base uranyl complexes that were prepared in this study have more effective IC50, which indicated less toxicity than previously reported results, which may be because this is the first study that investigated their effect against ovarian cancer and melanoma cell lines.

## 4. Conclusions

Schiff base ligands and their uranyl complexes were synthesized and characterized by analytical and spectroscopic methods. Geometric structures of the compounds were determined based on the results of FT-IR, ^1^H NMR, mass spectrometry, molar ratio, and thermal analysis. The IR spectra showed a new band in the region of 400 cm^−1^ for U-O and 500 cm^−1^ for U-N bonds, which indicates that the ligands coordinated with the uranyl ions through N, O bidentate binding in [UO_2_(L3)OAc]·2H_2_O and through N, N, O tridentate binding in [UO_2_(L1)_2_]·2H_2_O and [UO_2_(L2)OAc]·2H_2_O. From the IR spectra, the U-O bond lengths were calculated, and the results were compared with those from theoretical DFT calculations. Calculated data for [UO_2_(L2)OAc]·2H_2_O and [UO_2_(L3)OAc]·2H_2_O complexes indicated bidentate chelating of the acetate group, and this supports the results showing a molar ratio of 1:1. The acetate group does not appear in the [UO_2_(L1)_2_]·2H_2_O complex, which supports a metal/ligand molar ratio of 1:2. Thermal analysis indicated the number of water molecules in complexes, and conductivity showed that compounds were not electrolytes; as a result, it is concluded that complexes of Schiff base ligands and uranyl ions were formed.

DNA binding showed an intercalation mode of binding, with (K_b_) values in the range 1 × 10^−6^ to 3.33 × 10^−5^. Molecular docking analyses revealed that uranyl complexes have higher binding energies and scores than Schiff base ligands. Schiff base ligands and their uranyl complexes were examined against two types of cancer cell lines, and the data obtained suggested that the anticancer activities of uranyl complexes were higher than those of free ligands.

## Figures and Tables

**Table 1:** Validation of the basis set.

Basis Set		UO Bond Length, Å	% Error
For U Atom	For Other Atoms		
SDD	6-31G	1.451	17.73
SDD	6-31G(d,p)	1.280	27.38
SDD	6-31++G(d,p)	1.300	26.28
SDD	cc-pvdz	1.294	26.63
SDD	SDD	1.796	1.86

**Table 2:** The results of IR experimental and theoretical bands.

Compounds	(C=N) Azomethine		(C=N) Pyrazine Ring		C-O Phenol		C-X			
	Exp.	DFT	Exp.	DFT	Exp.	DFT	Exp.		DFT	
L1	1602	1621.94	1578.35	1588.34	1252.98	1289.55	630.59		634.76	
L2	1608.95	1798.16	1578.35	1668.00	1252.48	1354.52	ν_asym_	ν_sym_	ν_asym_	ν_sym_
							1559.70	1344.77	1897.89	1564.29
L3	1602.98	1676.97	1561.94	1573.69	1252.98	128529	------		------	
[UO_2_(L1)_2_]·2H_2_O	1639.91	1658.90	1612.68	1583.05	1255.97	1339.70	624.62		638.72	
[UO_2_(L2)OAc]·2H_2_O	1660.36	1660.32	1601.49	1585.48	1244.77	1348.61	ν_asym_	ν_sym_	ν_asym_	ν_sym_
							1542.50	1319.40	1471.66	1403.04
[UO_2_(L3)OAc]·2H_2_O	1637.69	1647.27	1558.95	1586.29	1250	1341.16	------		------	

X: L1 = Br, L2 = NO_2._

**Table 3:** (U-O) bond lengths (Å) in the IR experimental and DFT calculation.

Compounds	O=U=O				$r_{U-O}$	
	IR		DFT		IR	DFT
	ν_asym_	ν_sym_	ν_asym_	ν_sym_		
[UO_2_(L1)_2_]·2H_2_O	897.76	871.64	930.81	851.02	1.74 Å	1.79 Å
[UO_2_(L2)OAc]·2H_2_O	864.99	839.81	940.17	863.68	1.75 Å	1.79 Å
[UO_2_(L3)OAc]·2H_2_O	844.97	822.89	934.45	855.20	1.76 Å	1.79 Å

ν_asym_ = asymmetric, ν_sym_ = symmetric.

**Table 4:** Analytical and physical data of ligands and uranyl complexes.

Compounds	M.wt	Color	Yield %	M.p °C	Conductivity	Elemental Analysis			
						Found% (Calc.%)			
						C	H	N	O
L1	278.11	Orange	82.91	149.6–151.4	14	47.49 (47.51)	2.85 (2.90)	15.17 (15.11)	5.70 (5.75)
L2	244.21	Orange	93.12	210–212.7	19	53.89 (54.10)	3.20 (3.30)	22.90 (22.94)	19.69 (19.65)
L3	199.21	Orange	47	95–100.8	11	66.05 (66.32)	4.43 (4.55)	21.14 (21.09)	8.37 (8.03)
[UO_2_(L1)_2_]·2H_2_O	824.23	brown	83.29	122–320	43.74	31.77 (32.06)	1.69 (1.71)	10.35 (10.20)	7.80 (7.76)
[UO_2_(L2)OAc]·2H_2_O	572.27	yellow	88	291–321	54.81	25.59 (25.67)	2.31 (2.32)	9.14 (9.21)	23.88 (23.67)
[UO_2_(L3)OAc]·2H_2_O	527.28	Light orange	69.6	204–390	26.56	27.65 (27.67)	2.77 (2.86)	7.69 (7.45)	19.53 (19.85)

**Table 5:** Thermoanalytical result of L1.

Compounds	TG Range (°C)	Mass Loss% Calc. (Found)	Assignment	Metallic Residue
L1	54.53–248.32248.32–680.62	73.71 (73.09)16.6 (16.92)	Loss of (HBr), (2NO), (NH_3_), 2(C_2_H_2_) and (CO)Loss of (CO)	5C

**Table 6:** Thermodynamic data of thermal decomposition of L1.

Compounds	Method	Stages	An (s^−1^)	ΔG (kJ mol^−1^)	ΔH (kJ mol^−1^)	ΔS (Jmol^−1^)	E (kJ mol^−1^)	R^2^
L1	CR	1st	5.97 × 10^3^	1.77 × 10^5^	9.22 × 10^4^	−1.77 × 10^2^	9.62 × 10^4^	0.99940
	HM		1.23 × 10^−2^	1.44 × 10^5^	6.45 × 10^3^	−2.86 × 10^2^	1.05 × 10^5^	0.99952
	Average		2.99 × 10^3^	1.61 × 10^5^	4.93 × 10^4^	−2.31 × 10^2^	5.33 × 10^4^	0.99946
	CR	2nd	4.57 × 10^1^	2.30 × 10^5^	5.95 × 10^4^	−2.21 × 10^2^	6.59 × 10^4^	0.98963
	HM		6.16 × 10^2^	2.26 × 10^5^	7.27 × 10^4^	−1.99 × 10^2^	7.91 × 10^4^	0.99247
	Average		3.31 × 10^2^	2.28 × 10^5^	6.61 × 10^4^	−2.10 × 10^2^	7.25 × 10^4^	0.99105

**Table 7:** Coast–Redfern and Horowitz–Metzger plots of L1.

Compounds	Steps	Coast–Redfern Method	Horowitz–Metzger Method
L1	1st	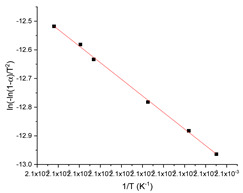	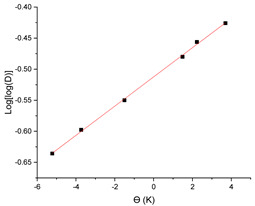
	2nd	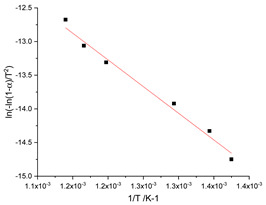	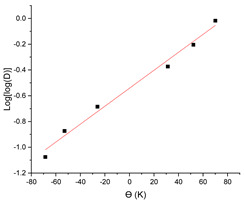

**Table 8:** The calculated quantum chemical parameters of the ligands and their uranyl complexes in (eV).

Quantum Chemical Parameters										
Compounds	HOMO	LUMO	ΔE	χ	η	*i*	σ	*S*	ω	ΔN_max_
L1	−6.25	−2.11	4.14	4.18	2.07	−4.18	0.48	0.24	4.22	2.02
L2	−6.59	−2.25	4.34	4.42	2.17	−4.42	0.46	0.23	4.50	2.04
L3	−6.24	−1.97	4.27	4.11	2.14	−4.11	0.47	0.23	3.95	1.92
[UO_2_(L1)_2_]·2H_2_O	−6.10	−3.18	2.92	4.64	1.46	−4.64	0.68	0.34	7.37	3.18
[UO_2_(L2)OAc]·2H_2_O	−7.02	−3.74	3.28	5.38	1.64	−5.38	0.61	0.30	8.82	3.28
[UO_2_(L3)OAc]·2H_2_O	−6.30	−3.23	3.07	4.77	1.54	−4.77	0.47	0.33	7.40	3.10

**Table 9:** The binding constant (K_b_) values of ligands and their uranyl complexes with CT-DNA.

Compounds	K_b_ (M^−1^)	Λmax Free (nm)	λ_max_ Bound (nm)	Type of Chromism
L1	1 × 10^6^	286	279 blue-shift	Hyperchromic
L2	6.67 × 10^5^	358	353 blue-shift	Hyperchromic
L3	8 × 10^5^	321	315 blue-shift	Hyperchromic
[UO_2_(L1)_2_]·2H_2_O	3.33 × 10^5^	317	312 blue-shift	Hyperchromic
[UO_2_(L2)OAc]·2H_2_O	4 × 10^5^	376	361 blue-shift	Hyperchromic
[UO_2_(L3)OAc]·2H_2_O	5 × 10^5^	327	322 blue-shift	Hyperchromic

**Table 10:** Docking score and energy of the ligands and uranyl complexes with 3W2S and 2OPZ protein cancer.

Types of Protein	Compounds	S	Rmsd_Refine	E_conf	E_place	E_refine	E_score2
Ovarian cancer *3W2S* protein	L1	−5.66	1.22	−6.44	−63.70	−10.96	−21.16
	[UO_2_(L1)_2_]·2H_2_O	−6.42	2.68	−3969.01	−19.27	−16.38	94.20
	L2	−5.54	1.65	5.19	−66.33	−11.20	−26.01
	[UO_2_(L2)OAc]·2H_2_O	−6.73	5.30	−2648.45	−71.11	−14.08	7.20
	L3	−5.40	0.98	53.32	−65.04	−9.60	−20.48
	[UO_2_(L3)OAc]·2H_2_O	−6.19	1.51	−1446.87	−41.42	−13.54	−19.76
Melanoma cancer *2OPZ* protein	L1	−6.03	1.43	−15.88	−48.82	−9.86	−21.53
	[UO_2_(L1)_2_]·2H_2_O	−6.68	2.53	−4015.41	−64.82	−12.28	−8.31
	L2	−6.14	3.84	6.52	−44.09	−9.28	−26.42
	[UO_2_(L2)OAc]·2H_2_O	−6.77	2.96	−2676.53	−26.45	−8.46	−4.73
	L3	−5.90	1.72	47.12	−60.04	−9.39	−18.68
	[UO_2_(L3)OAc]·2H_2_O	−6.41	6.21	−1566.02	−34.58	−9.59	−39.59

S = final score, which is the score of the last stage that was not set to any. Rmsd = root mean square deviation of the pose, in Å, from the original ligand. This field is present if the site definition was identical to the ligand definition. Rmsd_refine E = root mean square deviation between the pose before refinement and the pose after refinement. E_conf = the energy of the conformer. If there is a refinement stage, this is the energy calculated at the end of the refinement. Note that for forcefield refinement, by default, this energy is calculated with the solvation option set to born. E_place = score from the placement stage. E_score2 = score from rescoring stage 2. E_refine = score from the refinement stage, calculated to be the sum of the van der Waals electrostatics and solvation energies, under the generalized born solvation model (GB/VI).

**Table 11:** Interaction table between ligands and their uranyl (Π) complexes with 3W2S and 2OPZ proteins.

Types of Protein	Comp.	Ligand	Receptor	Interaction	Distance	E (kcal/mol)
Melanoma	**L1**	C 3	5-ring TRP 323 (A)	π-H	4.24	−0.8
		C 3	6-ring TRP 323 (A)	π-H	4.17	−0.8
		6-ring	CA LEU 307 (A)	π-H	4.80	−0.5
	[UO_2_(L1)_2_]·2H_2_O	O 2	OG1 THR 308 (A)	H-acceptor	3.16	−4.8
		6-ring	N THR 308 (A)	π-H	4.20	−1.7
		6-ring	CB THR 308 (A)	π-H	3.64	−0.6
	**L2**	O 24	N THR 308 (A)	H-acceptor	3.21	−1.7
		3-ring	CD LYS 297 (A)	π-H	3.81	−0.7
		6-ring	NZ LYS 297 (A)	π-cation	4.37	−3.0
	[UO_2_(L2)OAc]·2H_2_O	C 9	OE2 GLU 314 (A)	H-donor	3.37	−1.4
	**L3**	No measurable interactions				
	[UO_2_(L3)OAc]·2H_2_O	O 3	NZ LYS 322 (A)	H-acceptor	2.66	−7.9
		O 7	NZ LYS 322 (A)	H-acceptor	2.88	−3.4
		U 2	NZ LYS 322 (A)	Ionic	2.96	−4.8
		O 3	NZ LYS 322 (A)	Ionic	2.66	−7.2
Ovarian	**L1**	6-ring	CB ASP 837 (A)	π-H	3.75	−0.5
	[UO_2_(L1)_2_]·2H_2_O	C 15	OD1 ASP 837 (A)	H-donor	3.22	−1.2
		O 2	N GLY 724 (A)	H-acceptor	2.98	−26.8
	**L2**	C 11	NZ LYS 745 (A)	Ionic	3.21	−3.2
		6-ring	NZ LYS 745 (A)	π-cation	4.01	−0.6
	[UO_2_(L2)OAc]·2H_2_O	O 3	CA GLY 796 (A)	H-acceptor	3.27	−0.6
		O 5	CA GLY 719 (A)	H-acceptor	3.71	−1.3
	**L3**	6-ring	CB LYS 745 (A)	π-H	3.70	−1.0
	[UO_2_(L3)OAc]·2H_2_O	C 20	OD1 ASP 837 (A)	H-donor	3.28	−0.7
		N 19	NE ARG 841 (A)	H-acceptor	3.58	−0.7
		U 2	NZ LYS 745 (A)	Ionic	3.06	−4.1
		O 3	NZ LYS 745 (A)	Ionic	2.57	−8.1

**Table 12:** The docking model of Schiff base ligands and their uranyl complexes and the 3D and 2D snapshot.

		Melanoma *2OPZ* Result	Ovarian Cancer *3W2S* Result
L1	**2D**	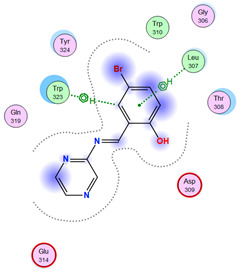	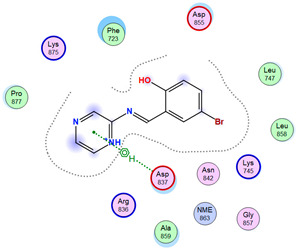
	**3D**	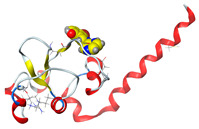	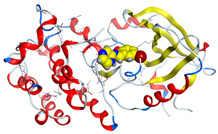
[UO_2_(L1)_2_]·2H_2_O	**2D**	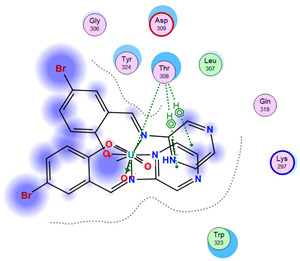	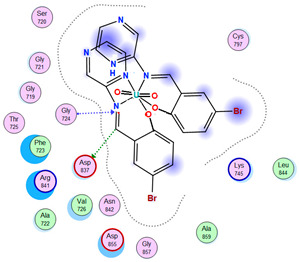
	**3D**	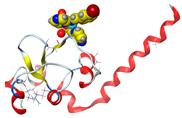	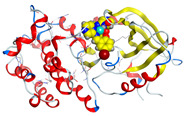
L2	**2D**	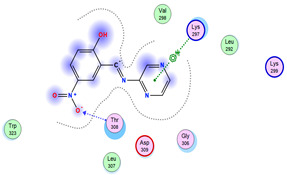	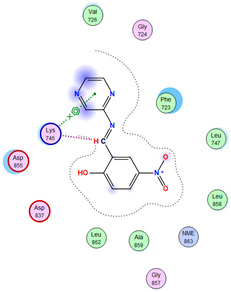
	**3D**	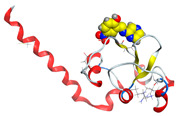	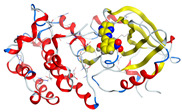
[UO_2_(L2)OAc]·2H_2_O	**2D**	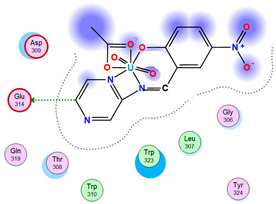	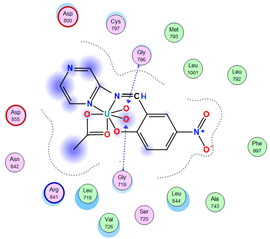
	**3D**	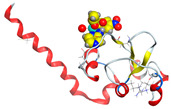	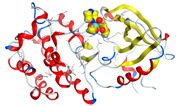
L3	**2D**	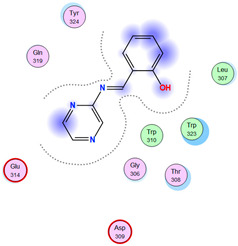	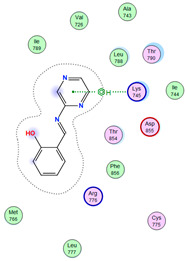
	**3D**	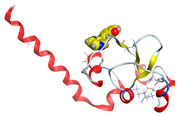	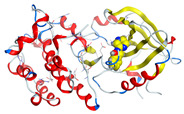
[UO_2_(L3)OAc]·2H_2_O	**2D**	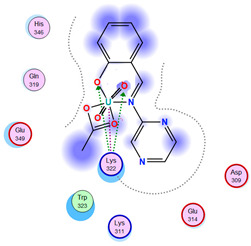	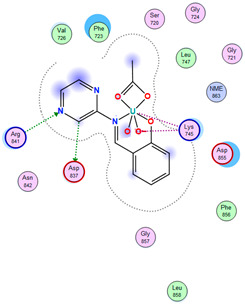
	**3D**	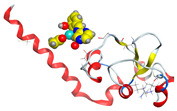	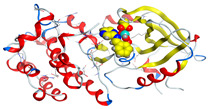
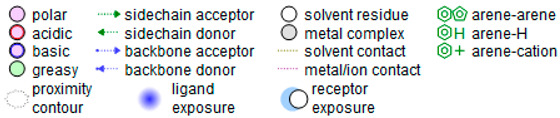			

**Table 13:** In vitro anticancer activity of Ovra3 and M14, two cancer cell lines.

Compounds	Ovar3 (Ovarian)		M14 (Melanoma)	
	IC50 μg/ml	SD	IC50 μg/ml	SD
L1	7.10	0.05	6.49	0.08
L2	7.01	0.05	6.35	0.08
L3	7.51	0.05	6.71	0.08
[UO_2_(L1)_2_]·2H_2_O	6.33	0.03	5.17	0.04
[UO_2_(L2)OAc]·2H_2_O	6.19	0.03	5.01	0.04
[UO_2_(L3)OAc]·2H_2_O	6.16	0.02	5.33	0.04

## Data Availability

All data availability is in supplementary data and any other data will be with corresponding author.

## Supplementary Materials

The following are available online at https://www.mdpi.com/article/10.3390/biom11081138/s1, Figure S1: Mass fragmentation pattern of L1, Figure S2: Mass fragmentation pattern of L2, Figure S3: Mass fragmentation pattern of L3, Figure S4: IR spectral for ligands and its uranyl complexes experimental, Figure S5: IR spectral for ligands and its uranyl complexes calculated, Figure S6: 1H NMR spectra of (L2 and L3), Figure S7: Mole ratio method plots of [UO2(L1)2]·2H_2_O and [UO2(L3)OAc]·2H_2_O complexes, Figure S8: TG curves for L1, L2, [UO2(L1)2]·2H_2_O and [UO2(L2)OAc]·2H_2_O, Figure S9: Absorption spectra of L2 in (Tris-HCl) buffer (pH = 7.4) at 25 °C with CT-DNA. The arrow indicates the increasing amount of DNA, Figure S10: Absorption spectra of L3 in (Tris-HCl) buffer (pH = 7.4) at 25 °C with CT-DNA. The arrow indicates the increasing amount of DNA, Figure S11: Absorption spectra of [UO2(L2)OAc]·2H_2_O in (Tris-HCl) buffer (pH = 7.4) at 25 °C with CT-DNA. The arrow indicates the increasing amount of DNA, Figure S12: Absorption spectra of [UO2(L3)OAc]·2H_2_O in (Tris-HCl) buffer (pH = 7.4) at 25 °C with CT-DNA. The arrow indicates the increasing amount of DNA, Table S1: Experimental data of [UO2(L3)OAc]·2H_2_O by molar ratio, Table S2: Electronic spectra results of ligands and its uranyl complexes experimental and calculated, Table S3: Thermoanalytical result of the ligands and its uranyl complexes, Table S4: Thermoanalytical result of the ligands and its uranyl complexes, Table S5: Thermodynamic data of thermal decomposition of ligands and its uranyl complexes, Table S6: Coast-Redfern and Horowitz-Metzger plots of ligands and its uranyl complexes.
