# Supplementary material for: Synthesis, Structural Investigations, Molecular Docking, and Anticancer Activity of Some Novel Schiff Bases and Their Uranyl Complexes
Source: Biomolecules. 2021 Aug 2;11(8):1138. doi: 10.3390/biom11081138 (PMC8391876; doi:10.3390/biom11081138)
Supplement: Supplementary file 1 [file biomolecules-11-01138-s001.zip › Hanan Uranyl complexes (Supplementary materials) 3.pdf]

Supplementary materials

Synthesis, structural investigations, molecular docking and anticancer activity of some novel Schiff bases and their Uranyl complexes

Hanan B.Howsaui [a], **Amal S.Basaleh** [a], **Magda H Abdellattif** [b*], **Walid M.I. Hassan** [a,c], **Mostafa A. Hussien** [a*,d]

[a] *Department of Chemistry, Faculty of Science, King Abdulaziz University, P.O. Box 80203 Jeddah 21589, Saudi Arabia.*

[b] *Department of chemistry College of Sciences Taif University, Al-Haweiah, P.O. Box 11099, Taif 21944, Saudi Arabia.*

[c] *Department of Chemistry, Faculty of Science, Cairo University, Giza, Egypt*

[d] *Department of Chemistry, Faculty of Science, Port Said University, Port Said 42521, Egypt*

[*] *email: maabdulaal@kau.edu.sa*

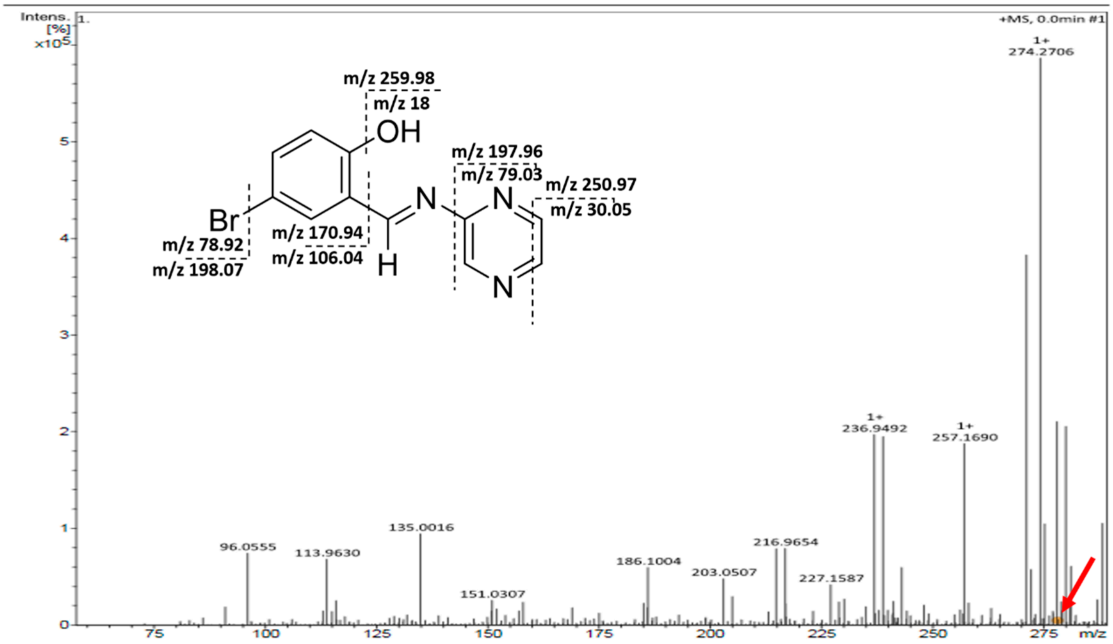

Figure S1. Mass fragmentation pattern of L1

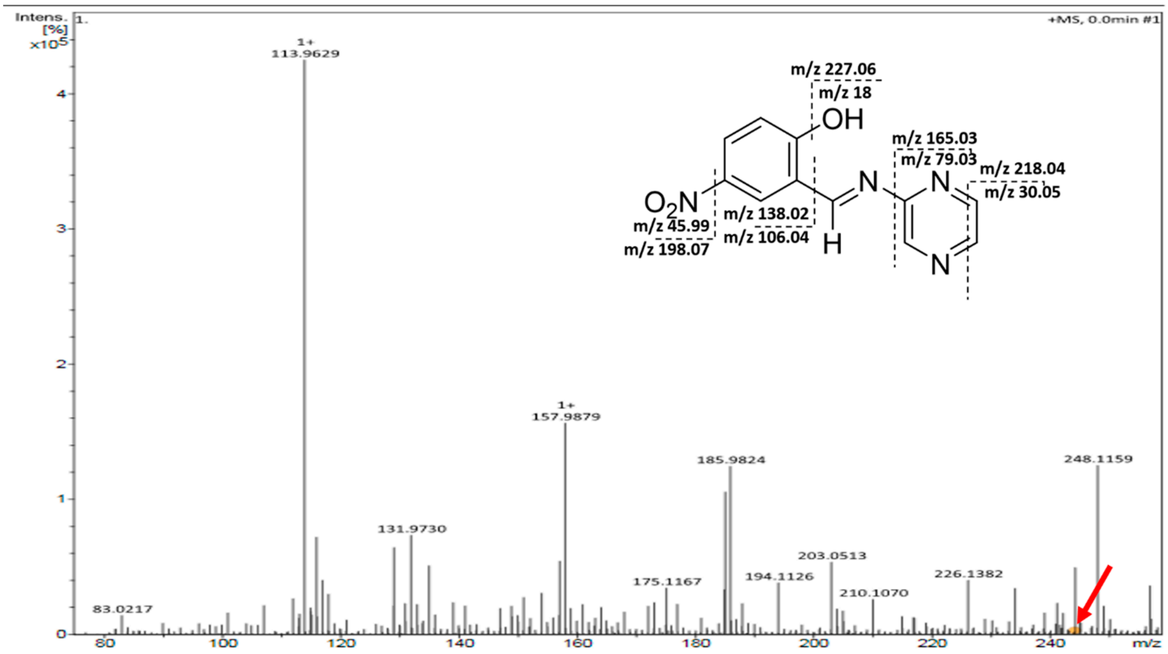

Figure S2. Mass fragmentation pattern of L2

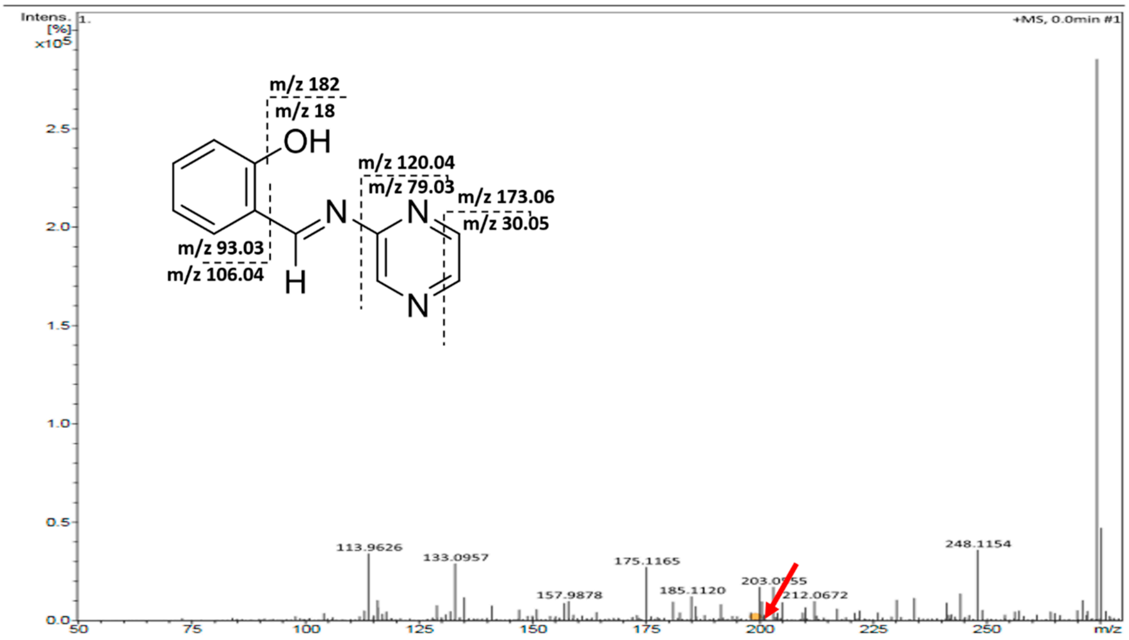

Figure S3. Mass fragmentation pattern of L3

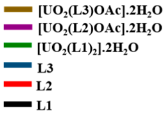

Figure S4. IR spectral for ligands and its uranyl complexes experimental

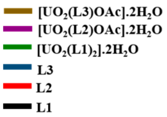

Figure S5. IR spectral for ligands and its uranyl complexes calculated

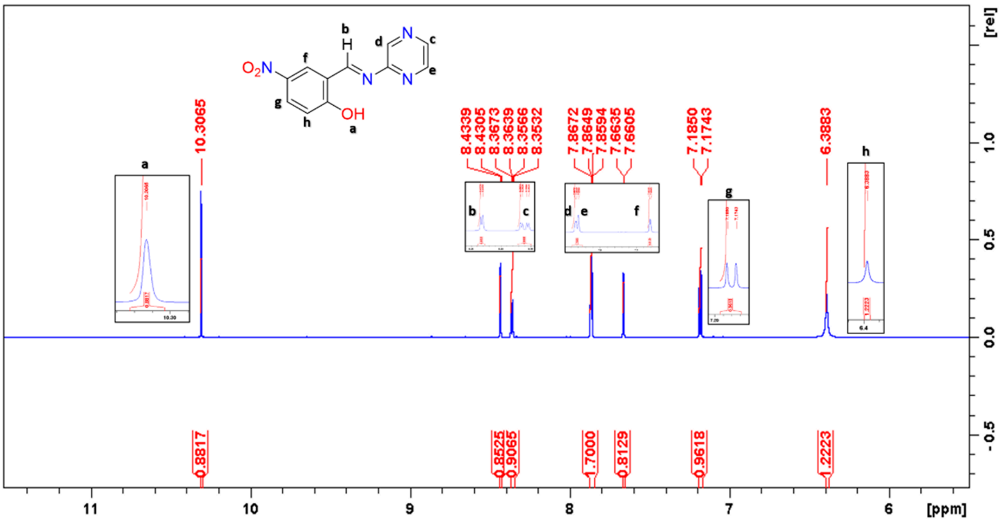

L2

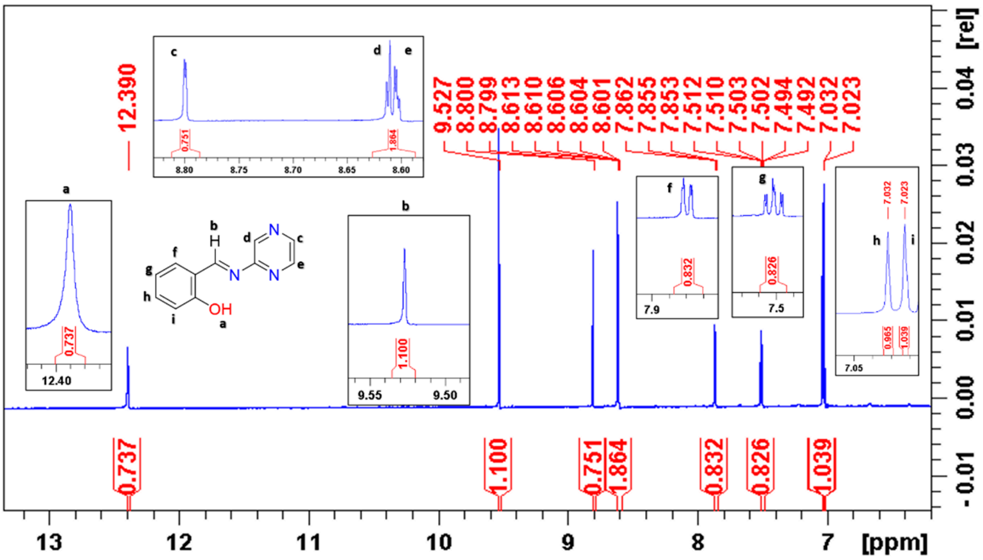

L3

Figure S6. ^{1}H NMR spectra of (L2, and L3)

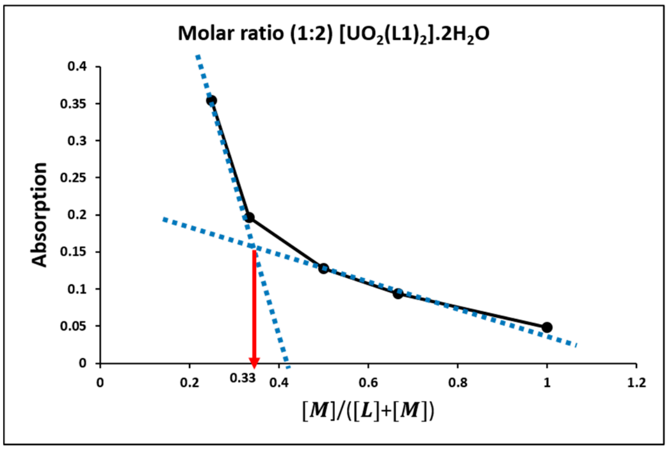

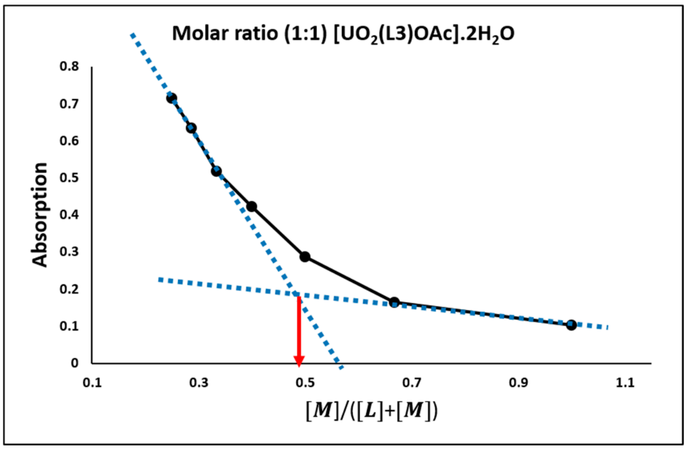

Figure S7. Mole ratio method plots of $[UO_2(L1)_2].2H_2O$ and $[UO_2(L3)OAc].2H_2O$ complexes

Table S1. Experimental data of $[UO_2(L3)OAc].2H_2O$ by molar ratio

	Metal C*10^{-4} moles	Ligand C*10^{-4} moles	[M]/([M]+[L])	Absorbance at 317_{nm}
1	0.72	2.16	0.25	0.71576
2	0.72	1.8	0.28	0.63353
3	0.72	1.44	0.33	0.51726
4	0.72	1.08	0.4	0.42197
5	0.72	0.72	0.5	0.28814
6	0.72	0.36	0.66	0.16332
7	0.72	0	1	0.10304

Table S2. Electronic spectra results of ligands and its uranyl complexes experimental and calculated

L1	Exp.	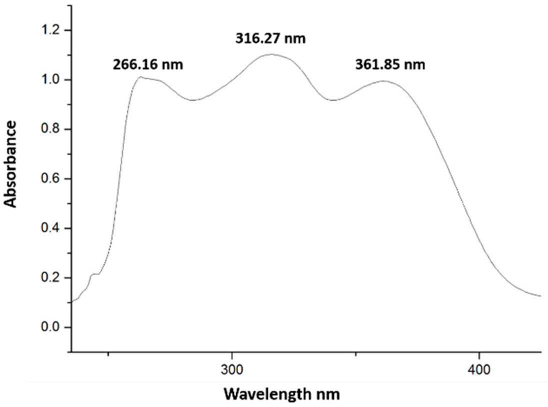
	DFT	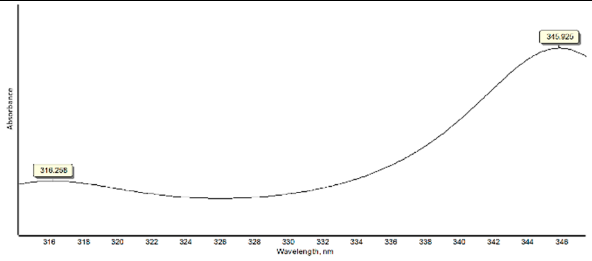
L2	Exp.	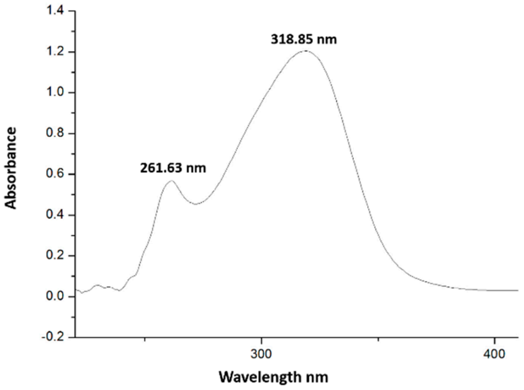
	DFT	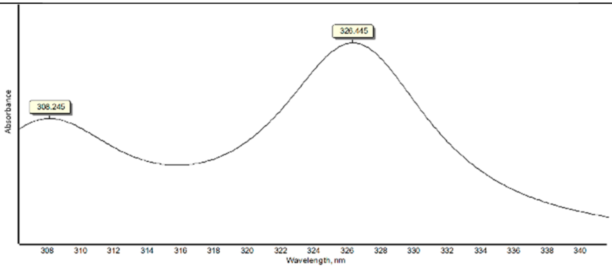

L3	Exp.	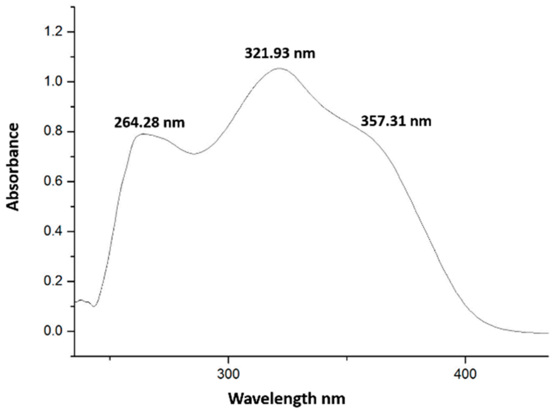
	DFT	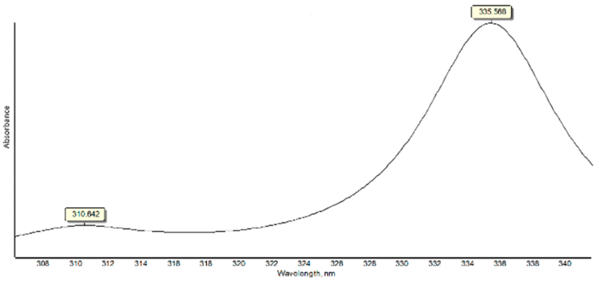
$[UO_2(L1)_2].2H_2O$	Exp.	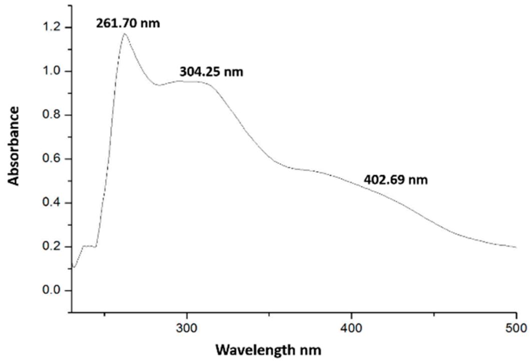
	DFT	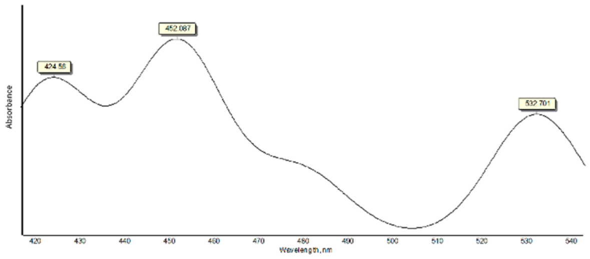
$[UO_2(L2)OAc].2H_2O$	Exp.	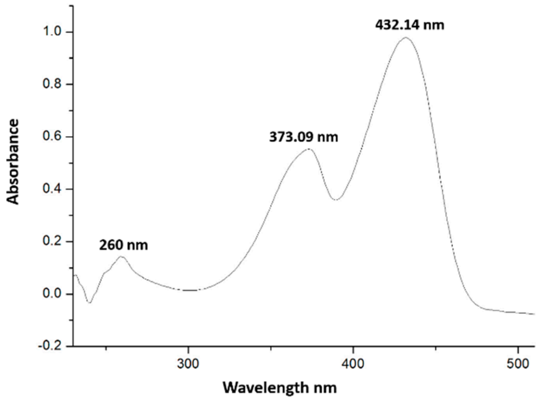

	DFT	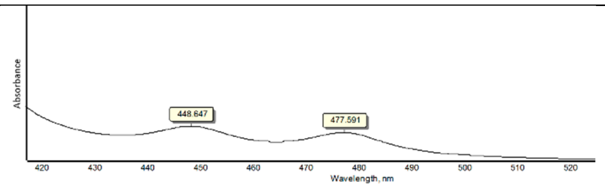
$[UO_2(L3)OAc].2H_2O$	Exp.	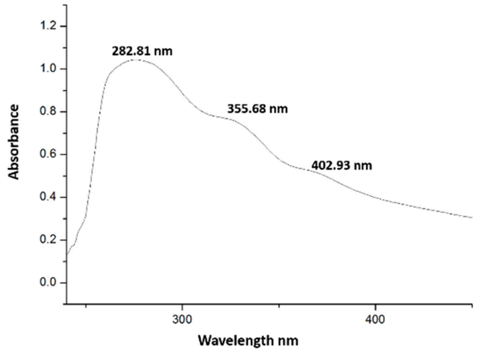
	DFT	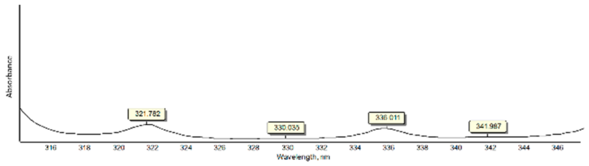

Table S3. Thermoanalytical result of the ligands and its uranyl complexes

Compounds	TG range (ºC)	Mass loss% calc. (Found)	Assignment	Metallic residue
L2	60.40-265.39<br>265.39-493.99	64.69 (64.84)<br>16.27 (16.18)	-Loss of $2(C_2H_2)$, (CH_4), (NO_2) and 2(NO)<br>-Loss of (NO)	6C
L3	57.09-237.30<br>237.30-798.68	65.25 (65.62)<br>24.65 (24.38)	-Loss of (H_2O), 2(NO) and $2(C_2H_2)$<br>-Loss of (NH_3)	7C
$[UO_2(L1)_2].2H_2O$	54.51-85.70<br>85.70-476.58<br>476.58-824.25	22.8 (22.7)<br>25.3 (25.9)<br>25.5 (24.5)	-Loss of $2(H_2O)$ and 2(HBr)<br>-Loss of 2(NO), $2(NH_3)$ and $3(C_2H_2)$<br>-Loss of 2(NO) and 8(CO)	$8C+O_3U$
$[UO_2(L2)OAc].2H_2O$	35.6-128.75<br>128.75-229.15<br>299.15-717.49<br>717.49-1000	14.2 (14.3)<br>6.6 (7.3)<br>29 (29.4)<br>14.1 (13.8)	-Loss of $5(NH_3)$ and (H_2O)<br>-Loss of 2(CO)<br>-Loss of 7(CO)<br>-Loss of 4(CO)	UO_3
$[UO_2(L3)OAc].2H_2O$	54-83<br>83-437<br>437-816	6.3 (5.5)<br>17.4 (17.1)<br>12.3 (12.4)	-Loss of $2(H_2O)$<br>-Loss of $2(NH_3)$, (CO) and (NO_2)<br>-Loss of (C_2H_2), (CO) and (CH_4)	$7C+O_3U$

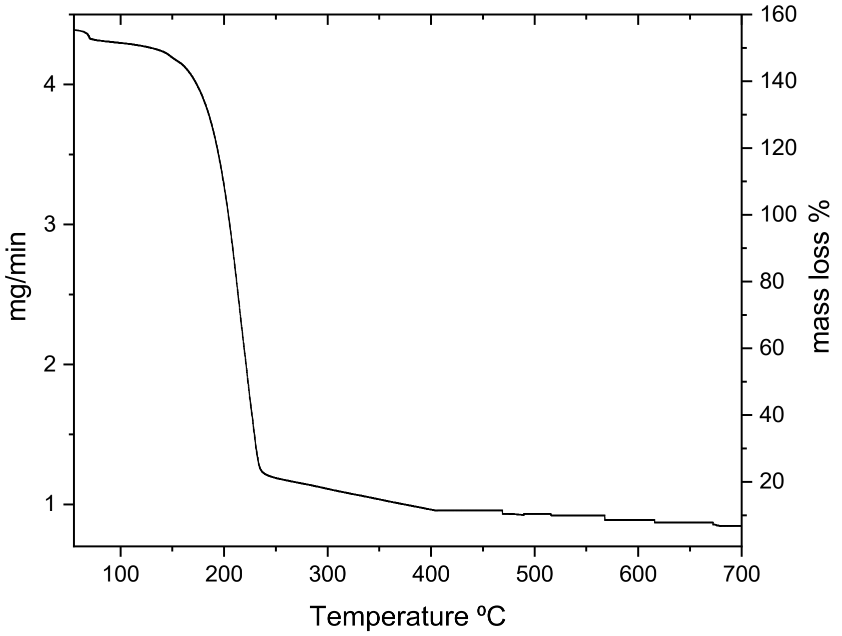

L1

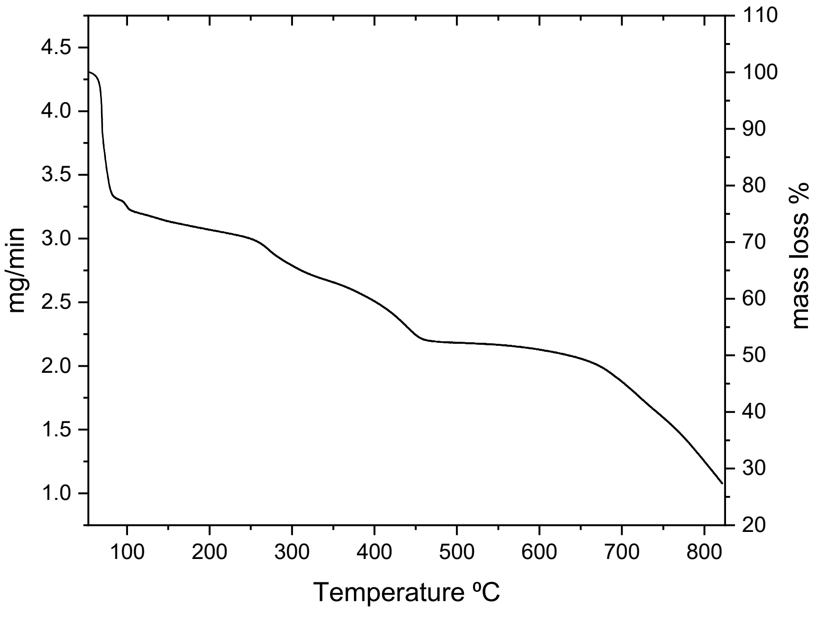

$[UO_2(L1)_2].2H_2O$

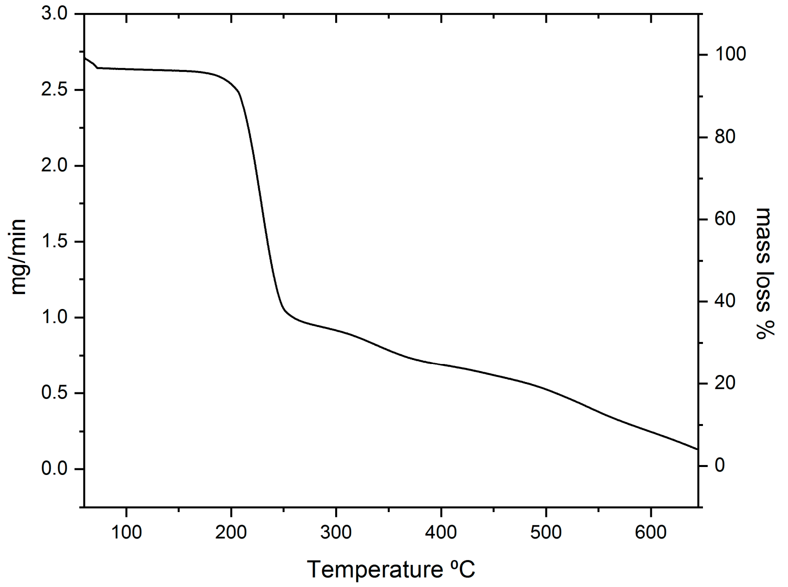

L2

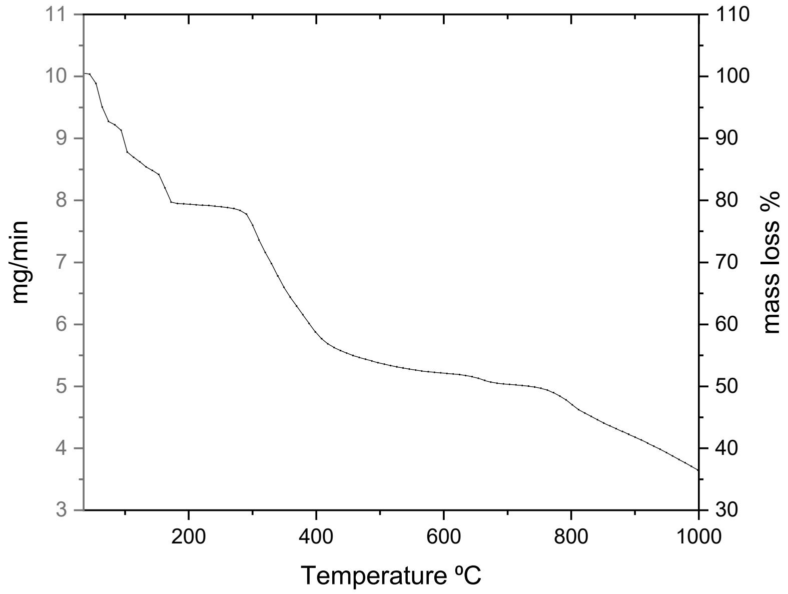

$[UO_2(L2)OAc].2H_2O$

Figure S8. TG curves for L1,L2, $[UO_2(L1)_2].2H_2O$ and $[UO_2(L2)OAc].2H_2O$

Table S4. Thermoanalytical result of the ligands and its uranyl complexes

Compounds	TG range (ºC)	Mass loss% calc. (Found)	Assignment	Metallic residue
L2	60.40-265.39<br>265.39-493.99	64.69 (64.84)<br>16.27 (16.18)	-Loss of 2(C_2H_2), (CH_4), (NO_2) and 2(NO)<br>-Loss of (NO)	6C
L3	57.09-237.30<br>237.30-798.68	65.25 (65.62)<br>24.65 (24.38)	-Loss of (H_2O), 2(NO) and 2(C_2H_2)<br>-Loss of (NH_3)	7C
[$UO_2(L1)_2$].2H_2O	54.51-85.70<br>85.70-476.58<br>476.58-824.25	22.8 (22.7)<br>25.3 (25.9)<br>25.5 (24.5)	-Loss of 2(H_2O) and 2(HBr)<br>-Loss of 2(NO), 2(NH_3) and 3(C_2H_2)<br>-Loss of 2(NO) and 8(CO)	8C+O_3U
[UO_2(L2)OAc].2H_2O	35.6-128.75<br>128.75-229.15<br>299.15-717.49<br>717.49-1000	14.2 (14.3)<br>6.6 (7.3)<br>29 (29.4)<br>14.1 (13.8)	-Loss of 5(NH_3) and (H_2O)<br>-Loss of 2(CO)<br>-Loss of 7(CO)<br>-Loss of 4(CO)	UO_3
[UO_2(L3)OAc].2H_2O	54-83<br>83-437<br>437-816	6.3 (5.5)<br>17.4 (17.1)<br>12.3 (12.4)	-Loss of 2(H_2O)<br>-Loss of 2(NH_3), (CO) and (NO_2)<br>-Loss of (C_2H_2), (CO) and (CH_4)	7C+O_3U

Table S5. Thermodynamic data of thermal decomposition of ligands and its uranyl complexes

Compounds	Method	Stages	A (s^{-1})	ΔG ($kjmol^{-1}$)	ΔH ($kjmol^{-1}$)	ΔS ($Jmol^{-1}$)	E ($kjmol^{-1}$)	R^2
L2	CR	1st	2.59×10^3	1.03×10^5	1.03×10^4	-1.84×10^2	1.45×10^4	0.99823
	HM		4.85×10^{-2}	1.49×10^5	1.12×10^4	-2.74×10^2	1.53×10^4	0.99876
	Average		1.30×10^3	1.26×10^5	1.07×10^4	-2.29×10^2	1.49×10^4	0.99849
	CR	2nd	2.55×10^2	1.57×10^5	2.35×10^4	-2.05×10^2	2.89×10^4	0.97225
	HM		2.34	1.92×10^5	3.32×10^4	-2.44×10^2	3.86×10^4	0.98776
	Average		1.29×10^2	1.74×10^5	2.84×10^4	-2.25×10^2	3.38×10^4	0.98000
L3	CR	1st	2.72×10^3	1.57×10^5	7×10^4	-1.38×10^2	7.39×10^4	0.99339
	HM		5.89×10^{-3}	1.42×10^5	7×10^4	-1.16×10^2	8.20×10^4	0.99437
	Average		1.36×10^3	1.49×10^5	4.26×10^3	-1.83×10^2	7.79×10^4	0.99388
	CR	2nd	6.23×10	2.86×10^5	3.71×10^4	-2.91×10^2	7.39×10^4	0.99339
	HM		1.39×10^2	2.82×10^5	7.99×10^4	-2.37×10^2	8.20×10^4	0.99437
	Average		7.29×10^1	2.84×10^5	6.99×10^4	-2.26×10^2	4.11×10^4	0.99388
[$UO_2(L1)_2$].2 H_2O	CR	1st	4.53×10^2	1.28×10^5	6.13×10^4	-1.95×10^2	6.42×10^4	0.99305
	HM		1.36×10^{-2}	1×10^5	4.09×10^3	-2.82×10^2	6.93×10^3	0.99388
	Average		2.26×10^2	1.14×10^5	3.27×10^4	-2.38×10^2	3.55×10^4	0.99346
	CR	2nd	9.66×10	1.33×10^5	8.69×10^3	-2.13×10^2	1.35×10^4	0.99041
	HM		1.43×10^{-1}	1.73×10^5	1.78×10^4	-2.67×10^2	2.27×10^4	0.99387
	Average		4.84×10	1.53×10^5	1.33×10^4	-2.40×10^2	1.81×10^4	0.99214
	CR	3ed	4.73×10^2	3.08×10^5	1.16×10^5	-2.03×10^2	1.24×10^5	0.99860
	HM		1.84×10^{-3}	2.97×10^5	6.09×10^3	-3.07×10^2	1.40×10^4	0.99881
	Average		2.37×10^2	3.03×10^5	6.09×10^4	-2.55×10^2	6.88×10^4	0.99870
[UO_2(L2)OA c].2H_2O	CR	1st	2.39×10^2	9.22×10^4	1.70×10^4	-2.01×10^2	2.01×10^4	0.97995
	HM		2.25	1.07×10^5	1.75×10^4	-2.40×10^2	2.06×10^4	0.98006

	Average		1.20×10^{2}	9.97×10^{4}	1.73×10^{4}	-2.21×10^{2}	2.04×10^{4}	0.98000
	CR	2nd	2.27×10^{2}	9.97×10^{4}	1.16×10^{4}	-2.03×10^{2}	1.52×10^{5}	0.99619
	HM		1.41×10^{-1}	1.27×10^{5}	1.23×10^{4}	-2.64×10^{2}	1.59×10^{4}	0.99591
	Average		1.13×10^{2}	1.13×10^{5}	1.20×10^{4}	-2.34×10^{2}	1.56×10^{4}	0.99605
	CR	3ed	4.68×10^{2}	1.62×10^{5}	3.54×10^{4}	-2×10^{2}	4.06×10^{4}	0.99100
	HM		4.41×10^{1}	1.85×10^{5}	4.60×10^{4}	-2.20×10^{2}	5.12×10^{4}	0.99395
	Average		2.56×10^{2}	1.73×10^{5}	4.07×10^{4}	-2.10×10^{2}	4.59×10^{4}	0.99247
	CR	4ed	1.04×10^{3}	3.31×10^{5}	7.70×10^{4}	-1.99×10^{2}	8.75×10^{4}	0.99821
	HM		4.35×10^{2}	3.85×10^{5}	1.22×10^{5}	-2.06×10^{2}	1.32×10^{5}	0.99773
	Average		7.37×10^{2}	3.58×10^{5}	9.93×10^{4}	-2.03×10^{2}	1.10×10^{5}	0.99797
$[UO_2(L3)OAc].2H_2O$	CR	1st	8.79×10^{2}	8.50×10^{4}	1.97×10^{4}	-1.90×10^{2}	2.25×10^{4}	0.99756
	HM		1.60×10	9.76×10^{4}	2.08×10^{4}	-2.23×10^{2}	2.37×10^{4}	0.98372
	Average		4.47×10^{2}	9.13×10^{4}	2.03×10^{4}	-2.06×10^{2}	2.31×10^{4}	0.99064
	CR	2nd	2.41×10^{1}	1.59×10^{5}	4.01×10^{4}	-2.23×10^{2}	4.46×10^{4}	0.99741
	HM		6.33×10^{2}	1.54×10^{5}	4.90×10^{4}	-1.96×10^{2}	5.34×10^{4}	0.99784
	Average		3.29×10^{2}	1.56×10^{5}	4.45×10^{4}	-2.10×10^{2}	4.90×10^{4}	0.99762
	CR	3ed	2.58×10^{2}	1.92×10^{5}	4.65×10^{3}	-2.08×10^{2}	1.21×10^{4}	0.99951
	HM		2.04×10^{-3}	2.81×10^{5}	6.04×10^{3}	-3.06×10^{2}	1.35×10^{5}	0.99671
	Average		1.29×10^{2}	2.25×10^{5}	5.36×10^{3}	-2.57×10^{2}	1.28×10^{4}	0.99811

Table S6 Coast-Redfern and Horowitz-Metzger plots of ligands and its uranyl complexes

compounds	steps	Coast-Redfern method	Horowitz-Metzger method
L1	1st	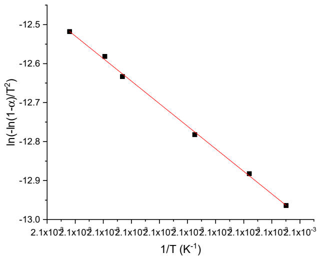	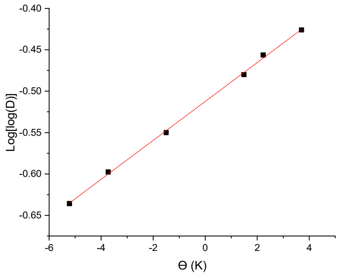
	2nd	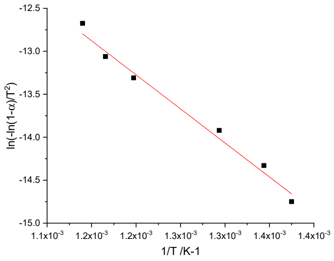	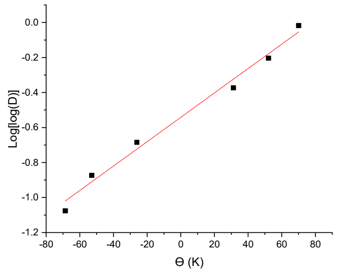

<table>
<tr><td rowspan="3">$[UO_2(L1)_2]$.
$2H_2O$</td><td>1st</td><td>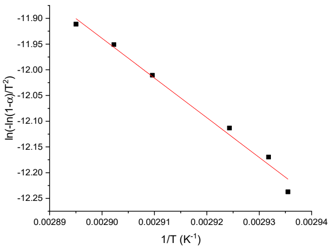
</td><td>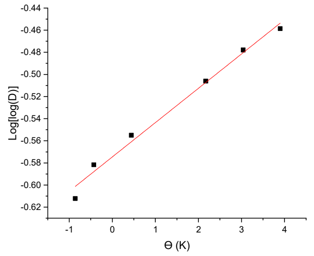
</td></tr>
<tr><td>2nd</td><td>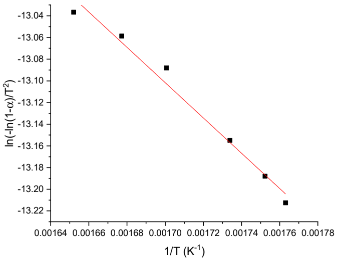
</td><td>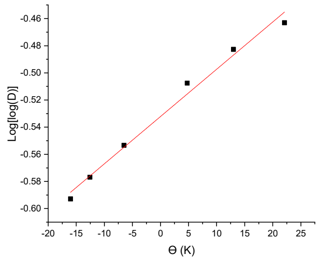
</td></tr>
<tr><td>3ed</td><td>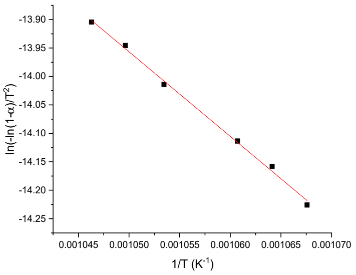
</td><td>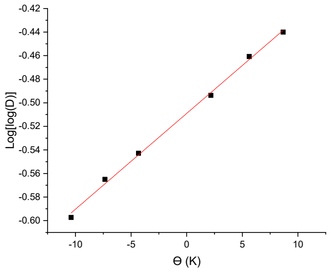
</td></tr>
<tr><td rowspan="2">L2</td><td>1st</td><td>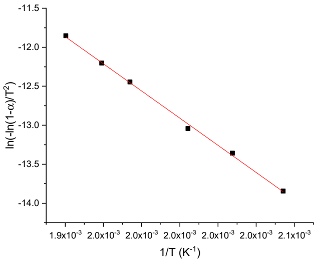
</td><td>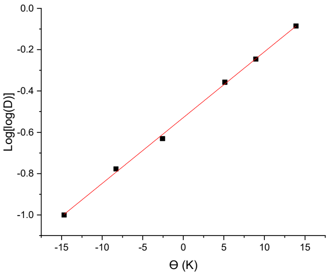
</td></tr>
<tr><td>2nd</td><td>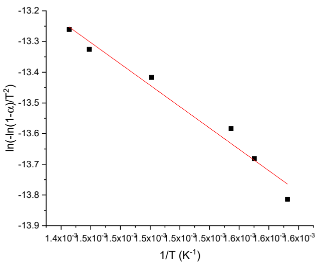
</td><td>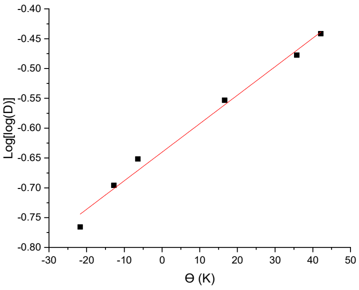
</td></tr>
</table>

<table>
<tr><td rowspan="4">[UO2(L2)OAc].2H2O</td><td>1st</td><td>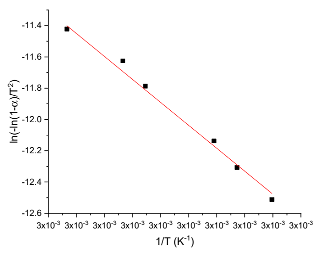
</td><td>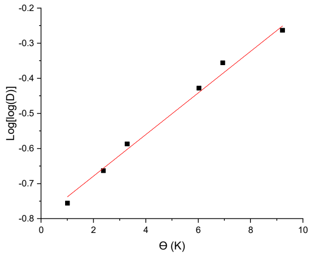
</td></tr>
<tr><td>2nd</td><td>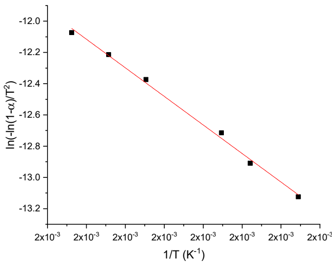
</td><td>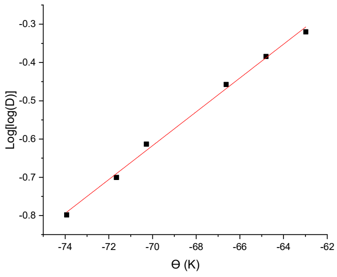
</td></tr>
<tr><td>3ed</td><td>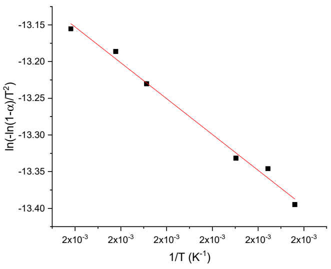
</td><td>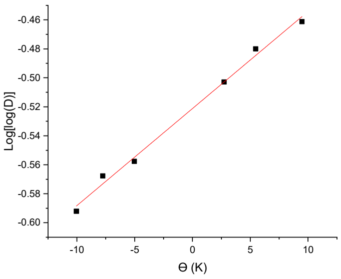
</td></tr>
<tr><td>4th</td><td>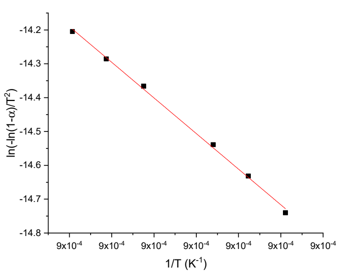
</td><td>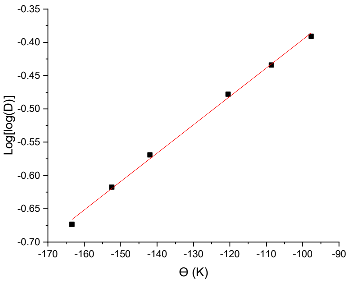
</td></tr>
<tr><td>L3</td><td>1st</td><td>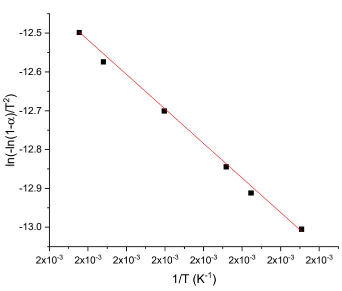
</td><td>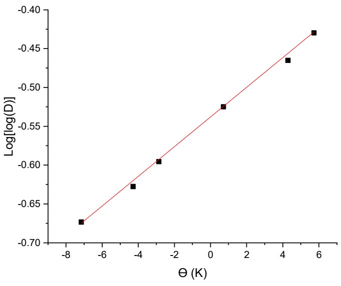
</td></tr>
</table>

	2nd	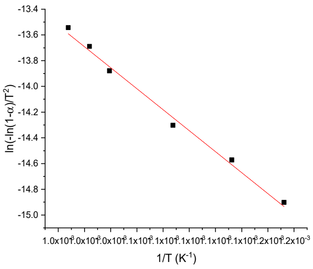	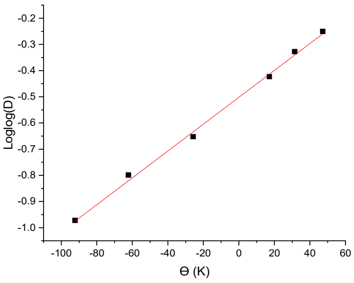
	1st	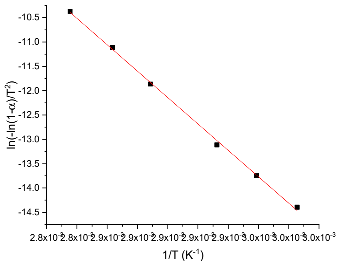	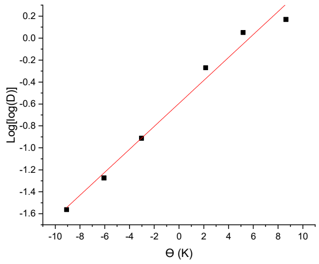
[UO_2(L3)OAc].$2H_2O$	2nd	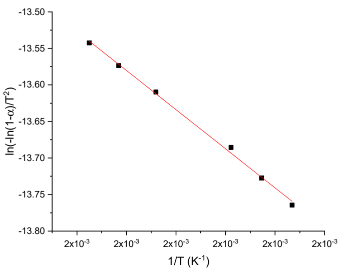	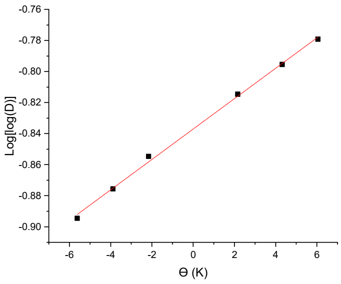
	3ed	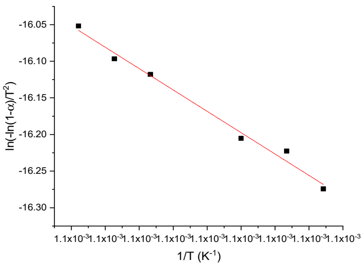	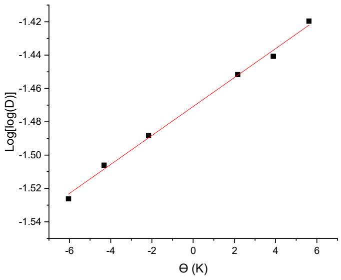

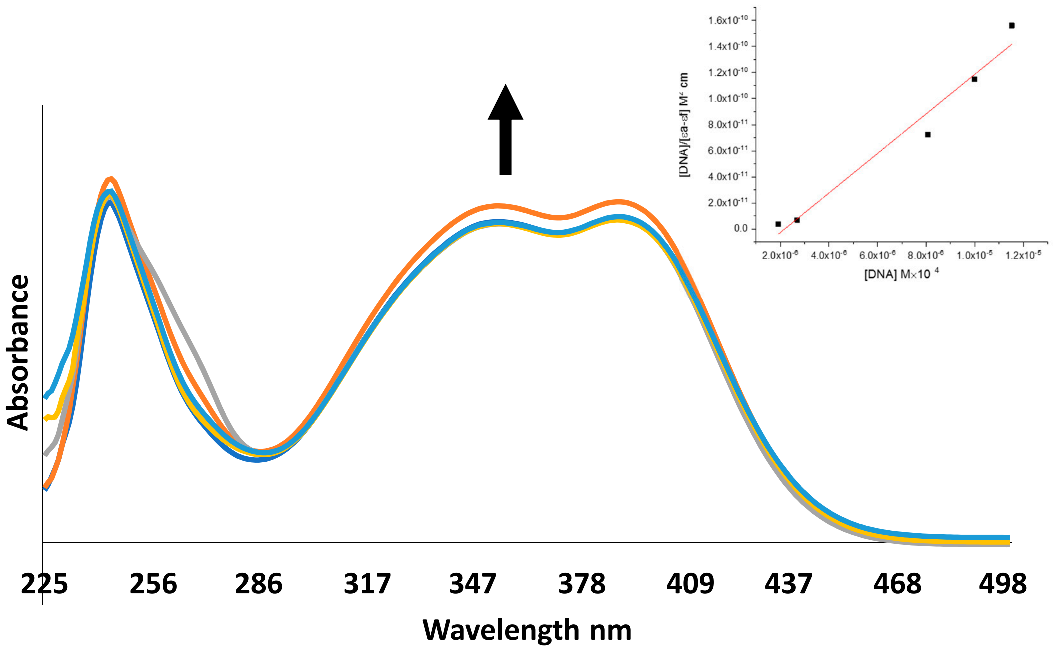

Figure 19. Absorption spectra of L2 in (Tris-HCl) buffer (pH=7.4) at 25 ºC with CT-DNA. The arrow indicates the increasing amount of DNA

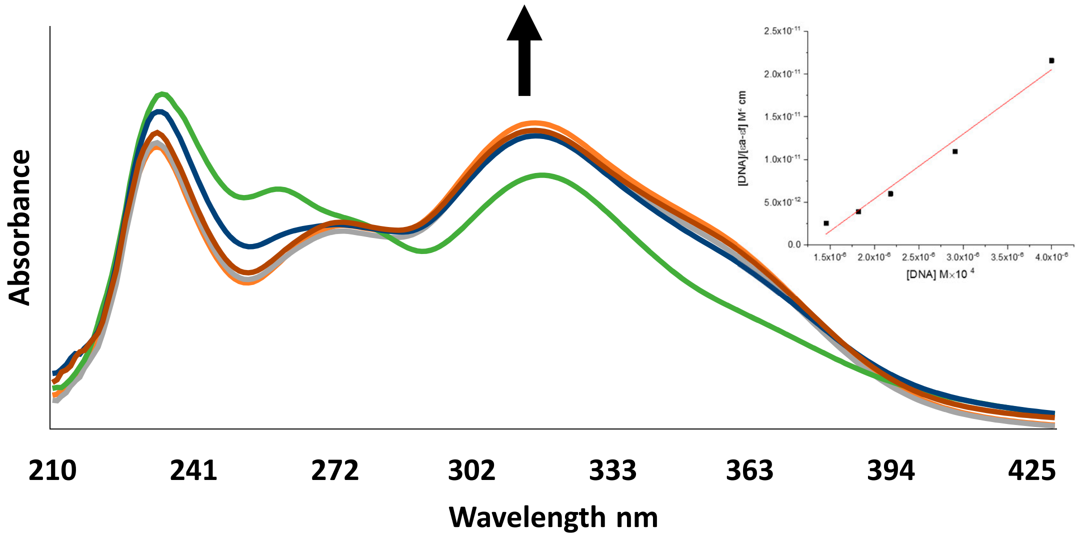

Figure 210. Absorption spectra of L3 in (Tris-HCl) buffer (pH=7.4) at 25 ºC with CT-DNA. The arrow indicates the increasing amount of DNA

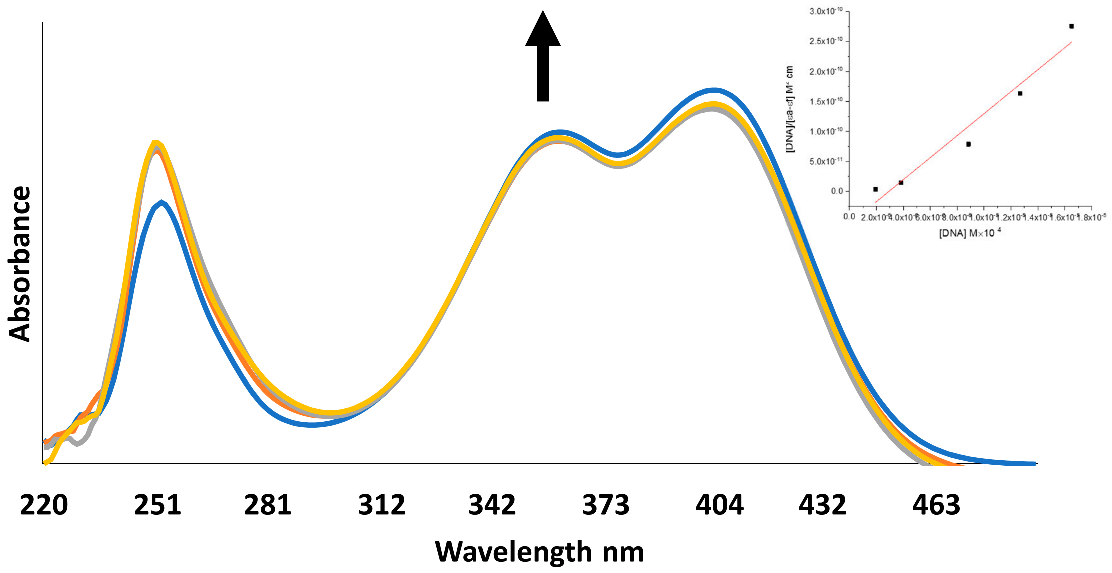

Figure 311. Absorption spectra of $[UO_2(L2)OAc].2H_2O$ in (Tris-HCl) buffer (pH=7.4) at 25 ºC with CT-DNA. The arrow indicates the increasing amount of DNA

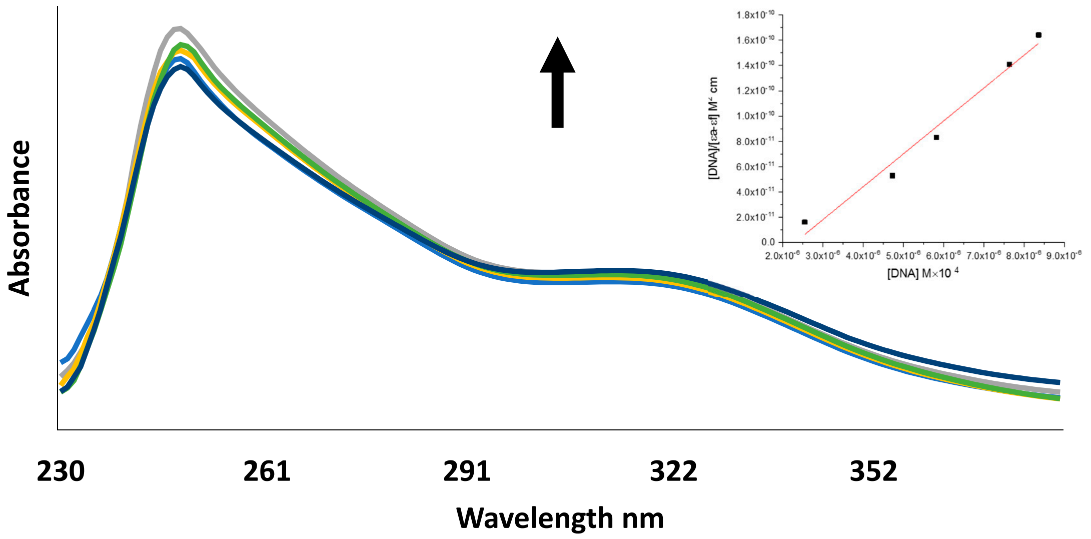

Figure 412. Absorption spectra of $[UO_2(L3)OAc].2H_2O$ in (Tris-HCl) buffer (pH=7.4) at 25 ºC with CT-DNA. The arrow indicates the increasing amount of DNA
